# Potentially Toxic Elements Accumulation and Health Risk Evaluation in Different Parts of Traditional Chinese Medicinal Materials

**DOI:** 10.3390/toxics14010040

**Published:** 2025-12-30

**Authors:** Jie Pan, Di Huang, Xue Ma, Di Zhu, Yuan Lu, Chunhua Liu, Lin Zheng, Yongjun Li, Jia Sun

**Affiliations:** 1 State Key Laboratory of Discovery and Utilization of Functional Components in Traditional Chinese Medicine, Engineering Research Center for the Development and Application of Ethnic Medicine and TCM (Ministry of Education), Guizhou Provincial Engineering Research Center for the Development and Application of Ethnic Medicine and TCM, Guizhou Medical University, Guian New Area 561113, China; 2 Guizhou Key Laboratory of Modern Traditional Chinese Medicine Creation, Guiyang 550004, China; 3 School of Pharmacy, Guizhou Medical University, Guian New Area 561113, China

**Keywords:** heavy metals and toxic elements, plants-derived materials, animal-derived materials, multivariate statistical analysis, source apportionment, ecological risk assessment, uncertainty analysis

## Abstract

This study systematically analyzed commercially available traditional Chinese medicines for As, Hg, Pb, Cd, and Cu, classifying them into roots and rhizomes (underground parts), stems and leaves, whole herbs, flowers, fruits and seeds (aboveground parts), and animal-derived decoction pieces. The concentration ranges of five elements in underground parts were 0~7.09, 0~0.29, 0~4.1, 0~1.1 and 0~49.2 mg/kg, with exceedance rates of 0–2.3%. Aboveground parts showed ranges of 0~1.54, 0~1.02, 0~13, 0~0.96 and 0~43.4 mg/kg, with exceedance rates of 0–8.8%. Animal-derived decoction pieces showed ranges of 0.07~27.18, 0~1, 0~55, 0~4.11 and 0.23~43.9 mg/kg, with exceedance rates of 6.7–41.3%. Principal component and cluster analyses indicated distinct contamination sources between animal-derived and plant-derived materials. The pollution index showed that animal-derived materials required special attention. Among plant-derived materials, *Notoginseng Radix et Rhizoma*, and *Artemisiae Argyi Folium* were also of concern. Health risk assessment indicated low non-carcinogenic risks across all categories (*HI* < 1), and uncertainty analysis showed a 0% probability of *HI* > 1. The 95th percentile carcinogenic risk for all categories was <1 × 10^−4^. Sensitivity analysis identified metal concentrations and daily intake as key uncertainty contributors. The findings underscore distinct contamination patterns between material types, highlighting the need for targeted control strategies, including strengthened source management and standardized dosing.

## 1. Introduction

In recent years, the international recognition and influence of Traditional Chinese Medicinal Materials (TCMMs) have increased significantly. The export of these materials to countries such as Japan, Vietnam, and Thailand has grown steadily, and Chinese herbal resources are now widely used around the world [1,2]. According to the World Health Organization (WHO), approximately 4 billion people worldwide currently use herbal medicine, with up to 80% of the global population having used TCMMs at some point [3]. Concurrently with this growth in demand and influence, concerns regarding its application safety have intensified. China, being the principal origin of the international trade of these materials, should ensure their safety quality. However, during cultivation, processing, transportation, and storage, TCMMs may become contaminated by exogenous harmful substances, which can persist in the product and pose potential risks to human health [4]. Exogenous harmful substances primarily include potentially toxic elements (PTEs), pesticide residues, sulfur dioxide, and mycotoxins [5,6]. Pesticide residues and sulfur dioxide problems primarily arise from human application, while mycotoxins are mainly caused by improper conditions during harvesting and processing. The sources of contamination by PTEs are more extensive. With industrial development, human activities such as urban emissions and the use of agricultural fertilizers have become the primary sources of pollution for these elements [7,8]. Therefore, compared to other exogenous harmful substances, contamination by PTEs is more challenging to control during cultivation, processing, and storage.

A body of evidence suggests that exogenous contaminants, including arsenic (As), mercury (Hg), lead (Pb), cadmium (Cd), and copper (Cu), may pose potential risks to human health. These elements have been reported to enter the body through various pathways such as ingestion, dermal absorption, and bioaccumulation in the food chain [9]. Excessive exposure to PTEs can lead to cancer, cardiovascular diseases, neurodegenerative disorders, and multi-organ damage [10,11]. Specifically, excessive lead intake can damage the kidneys and nervous system [12]. Overconsumption of As increases the risk of cardiovascular diseases, neurological disorders, skin cancer, as well as cancers of internal organs and lungs [13]. Cd accumulation can lead to liver and kidney damage, as well as osteoporosis [14,15]. Chronic exposure to methyl-mercury (MeHg) can severely damage the cerebral and cerebellar cortices, leading to central nervous system dysfunction [16]. Although Cu is an essential element, excessive intake can lead to dermatitis, upper respiratory irritation, gastrointestinal distress, and liver damage [17].

According to raw materials and medicinal parts, TCMMs can be divided into three types: (1) roots and rhizomes of plants (underground parts), (2) stems and leaves, whole herbs, flowers, fruits and seeds (aboveground parts), and (3) animal-derived decoction pieces. Animal-derived decoction pieces refer to medicinal products derived from animals or their by-products, processed for use in prescriptions. They have a long history of use in TCMMs, and compared to plant-based decoction pieces, animal-derived decoction pieces are more prone to bioaccumulation and biomagnification effects in the food chain [18]. Existing research shows that animal-derived decoction pieces have a high bioaccumulation potential for PTEs [19,20,21,22].

At present, the Contamination of PTEs has become a key issue threatening the quality of Chinese medicinal materials, edible spices and other products and human health. Djahed, B., Rasic Misic, I.D. and others have carried out research on the PTE pollution and risk assessment of edible spices and TCMMs, clarifying the core characteristics of pollution and health risks [23,24]. A global systematic review showed that As, Pb, Cd, and Cu are frequently detected in spices and herbal plants. The carcinogenic risk (*CR*) or non-carcinogenic risk quotient (*THQ*) of some regions and varieties has exceeded the acceptable level, highlighting the necessity of controlling PTE pollution in food and medicinal plants globally [25]. Research on plant-derived medicinal materials in China further indicates that PTE residues are closely linked to human activities. As is the main non-carcinogenic risk element, and its risk distribution shows a pronounced north–south polarization [26]. However, these studies have not included animal-derived decoction pieces and have not systematically compared contaminant levels across medicinal parts (underground vs. Aboveground part). Existing health risk assessments also have not considered the randomness of contaminant concentration distribution and human exposure parameters, making it difficult to reduce assessment uncertainty. Research by Mirzabayati, F. and Liu Y. has expanded to all types of TCMMs (including medicinal plants, animal-derived medicines, and mineral-derived medicines), focusing on the influencing factors of PTE accumulation. Findings show that accumulation is largely driven by the growth environment (soil and water), species characteristics, medicinal part, and human activities such as harvesting, processing, and storage [27,28]. Although some studies cover multiple categories of TCMMs, systematic comparisons of contamination across medicinal parts and animal-derived materials, as well as comprehensive health risk assessments, are still lacking.

Therefore, to comprehensively compare contaminant level differences across medicinal parts and animal-derived decoction pieces, identify potential sources, and evaluate health risks, this study selected As, Hg, Pb, Cd, and Cu as target pollutants. Element concentrations were measured in roots and rhizomes (underground parts), stems and leaves, whole herbs, flowers, fruits and seeds (aboveground parts), as well as animal-derived decoction pieces, followed by comparative analyses. Principal component analysis (PCA) and hierarchical cluster analysis (HCA) were applied to assess contamination patterns and identify potential sources. In addition, the single-factor pollution index (*P_i_*) and Nemerow pollution index (*P_N_*) were used to evaluate contamination levels, and health risk assessment models were employed to calculate non-carcinogenic and carcinogenic risks. To further reduce the uncertainty inherent in traditional assessments and enhance the reliability of the results, a probabilistic model incorporating contaminant concentration distributions and human exposure parameters was introduced. This study provides an important basis for establishing scientific limit standards, identifying high-risk medicinal materials, and ensuring medication safety.

## 2. Materials and Methods

### 2.1. Sample Collection

**Figure 1:**
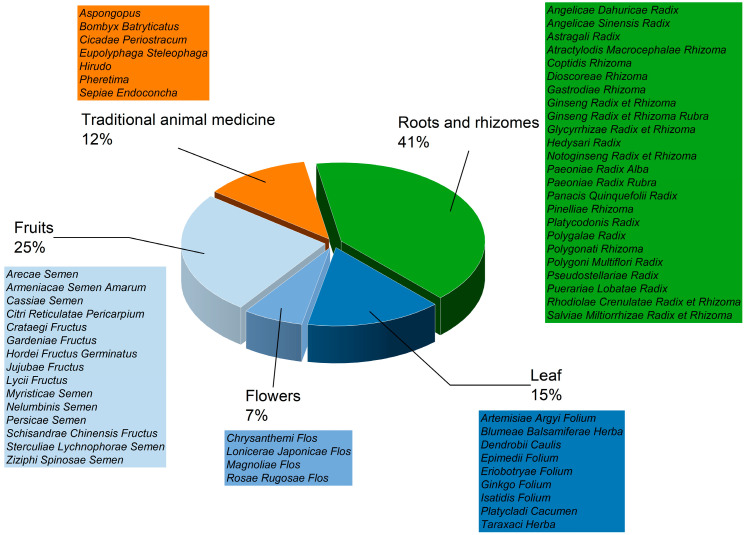
Summary of sample information for 422 batches from 59 species.

A total of 422 batches of 59 types of TCMMs were collected via both online and offline channels, including 24 root and rhizome types, 9 stem and leave, whole herb types, 4 flower types, 15 fruit and seed types, and 7 animal medicine types. Among these, 75 batches were animal-based, and 347 batches were plant-based (including 177 root-based, 47 stem, leaf, herb-based, 29 flower-based, and 94 fruit and seed-based). Sample classification information is shown in Figure 1 and Table S4.

### 2.2. Sample Processing and Analysis

After collection, all TCMMs were stored in a cool and dry place. The samples were then dried at 60 °C and ground into powder, and each batch of samples was accurately weighed into three replicates of 0.5 g and placed into digestion vessels (polytetrafluoroethylene inner liners, RNK Science and Technology, Nanjing, China). Each sample was digested with 7 mL of nitric acid in a sealed high-pressure digestion vessel heated at 170 °C for 3 h. After cooling, the solution was heated again at 170 °C on a hotplate to drive off the red-brown nitrogen oxide fumes until approximately 1 mL of liquid remained. The cooled digestate was transferred to a 50 mL volumetric flask, mixed with 2.5 mL of concentrated hydrochloric acid, and diluted to volume with water to obtain the final test solution. Pb and Cd detection was performed using graphite furnace atomic absorption spectrophotometry (GFAAS), Cu detection was performed using flame atomic absorption spectrophotometry (FAAS), and As and Hg were detected by Atomic Fluorescence Spectrometry (AFS). The experiment followed the general principles of Atomic Absorption Spectrophotometry (AAS) outlined in the Chinese Pharmacopoeia 2321, the instrument manual, and previously established laboratory protocols [6,29] to set the operating parameters of the atomic absorption spectrophotometer (PerkinElmer, Waltham, MA, USA). Specific parameters are listed in Table S1. The analysis of PTEs in this study followed the internal standard operating procedures previously validated in our research group [29,30]. The methodological results indicated that the recovery rates for the target PTEs fell within the acceptable range of 95.4% to 113.5%, and the relative standard deviation (RSD) between replicate samples was generally less than 8% (Tables S2-2, S2-3 and S3). These measures helped ensure the accuracy and reliability of the analytical results. To evaluate the accuracy of the analysis method, certified standard solutions of As (Batch No. 22B006-4), Hg (Batch No. 2209012-6), Pb (Batch No. 22A020-6), Cd (Batch No. 22B039-3), and Cu (Batch No. 22B015-2) were purchased from the National Center of Analysis and Testing for Nonferrous Metals and Electronic Materials, which were stored in nitric acid as stock solutions and diluted with either water or a 2% hydrochloric acid solution to prepare a series of mixed standard solutions at varying concentrations. These solutions were analyzed under the established instrument conditions, and calibration curves were constructed. The results demonstrated excellent linearity over a defined concentration range, with correlation coefficients (*R* ≥ 0.998) (Table S2-1). The limits of detection (LODs) were, respectively, 0.1 × 10^−3^ mg/kg for As, 0.03 × 10^−4^ mg/kg for Hg, 1.2 × 10^−4^ mg/kg for Pb, 0.3 × 10^−5^ mg/kg for Cd, and 0.02 mg/kg for Cu. All measured recovery rates fell within the acceptable range.

### 2.3. Analysis of PTEs Pollution Sources in Different Types of TCMMs

Based on the concentrations of the detected elements, PCA [31] and HCA [32] were used to examine the differences in PTE contamination among different medicinal parts and to explore their potential sources.

### 2.4. Assessment of PTEs Contamination Levels in Different Types of TCMMs

#### 2.4.1. The Single-Factor Pollution Index

The method is used to assess pollution levels by comparing the measured value to the permissible standard, using the following equation:

(1) $$P_{i}=\frac{C_{i}}{S_{i}}$$

where *P_i_* is the single-factor pollution index of the element, *C_i_* is the measured concentration, and *S_i_* is the standard limit for the element. These *S_i_* standard limits were adopted from the Chinese Pharmacopoeia and were set as follows: 2 mg·kg^−1^ for As, 0.2 mg·kg^−1^ for Hg, 5 mg·kg^−1^ for Pb, 1 mg·kg^−1^ for Cd, and 20 mg·kg^−1^ for Cu (excluding *Sepiae Endoconcha* and *Hirudo*). The standards for *Sepiae Endoconcha* are 10, 0.2, 5, 5, and 20 mg·kg^−1^, respectively. For Hirudo, the corresponding standards are 5, 1, 10, 1, and 20 mg·kg^−1^ [6]. Based on the *P_i_* standard, the following categories can be established: *P_i_* ≤ 1, non-pollution; 1 < *P_i_* ≤ 2, slight pollution; 2 < *P_i_* ≤ 3, moderate pollution; *P_i_* > 3, heavy pollution [33].

#### 2.4.2. The Nemerow Pollution Index

The *P_N_* combines the *P_i_* evaluation with environmental quality indices that consider both extreme values and average values. The formula is:

(2) $$P_{N}=\sqrt{\frac{P_{iave}^{2}+P_{imax}^{2}}{2}}$$

where *P_iave_* is the average value of *P_i_* of all PTEs and *P_max_* is the maximum value. *P_N_* can be classified as follows: *P_N_* ≤ 0.7, safe level; 0.7 < *P_N_* ≤ 1, alert level; 1 < *P_N_* ≤ 2, slight pollution; 2 < *P_N_* ≤ 3, moderate pollution; *P_N_* > 3, heavy pollution [34].

### 2.5. Health Risk Assessment

*THQ* and *CR* are methods proposed by the United States Environmental Protection Agency (USEPA) to assess the risks associated with human exposure to PTEs through dietary intake [35]. These approaches can be applied to assess the non-carcinogenic and carcinogenic risks associated with contaminant levels in specific foods, medicinal herbs, or formulations under defined consumption doses [5].

#### 2.5.1. Non-Carcinogenic Risks

The present study evaluated both the non-carcinogenic and lifetime carcinogenic risks associated with specific PTEs in TCMMs. Given that TCMMs are typically administered as water decoctions, the transfer rates of PTEs during the decoction process, as reported by [36,37], were incorporated into the calculations. Non-carcinogenic health risks were assessed using the following equation:

(3) $$\mathrm{THQ}=\frac{\mathrm{EF}\times \mathrm{ED}\times \mathrm{IRD}\times \mathrm{Ci}\times t}{\left(\mathrm{RfD}\times \mathrm{BW}\times \mathrm{AT}\right)}\times 10^{-3}$$

*THQ* represents the Target Hazard Quotient, *EF* is the exposure frequency (90 days/year), *ED* stands for the exposure duration (20 years), *C_i_* (mg·kg^−1^) is the concentration of the element in decoction pieces, *IRD* denotes the daily consumption amount of the medicinal material, following the average dosage outlined in the 2025 Chinese Pharmacopoeia [6], *t* is the transfer rate of PTEs after water decoction (35% for As, 24% for Hg, and 14% for Pb, Cd, and Cu) [36], *RfD* stands for the oral reference dose as recommended by USEPA: 0.0035, 0.001, 0.0003, 0.0003, and 0.0400 mg·kg^−1^·d^−1^) for Pb, Cd, Hg, As, and Cu [38,39], respectively, *BW* is the average body weight of an adult (60 kg), and *AT* is the average lifespan (365 days/year × 70) [40].

Due to the cumulative risk posed by multiple PTEs present in the same decoction piece, the Hazard Index (*HI*) was therefore employed to evaluate the non-carcinogenic risk, which is calculated as follows:

(4) $$\mathrm{HI}={\mathrm{THQ}}_{\mathrm{AS}}+{\mathrm{THQ}}_{\mathrm{Pb}}+{\mathrm{THQ}}_{\mathrm{Cd}}+{\mathrm{THQ}}_{\mathrm{Hg}}+{\mathrm{THQ}}_{\mathrm{Cu}}$$

#### 2.5.2. Carcinogenic Risk


(5) $$\mathrm{CR}=\frac{\mathrm{EF}\times \mathrm{ED}\times \mathrm{IRD}\times \mathrm{Ci}\times t\times \mathrm{CSF}}{\mathrm{BW}\times \mathrm{AT}}\times 10^{-3}$$

*CR* represents the carcinogenic risk factor (USEPA considers an acceptable carcinogenic risk level to range from 1 × 10^−6^ to 1 × 10^−4^; values exceeding 1 × 10^−4^ indicate a significant carcinogenic risk) [12,41]. *CSF* refers to the carcinogenic slope factor, as defined in the USEPA’s carcinogenic risk assessment guidelines: 1.5 (mg·kg^−1^·d^−1^) for As, 0.0085 (mg·kg^−1^·d^−1^) for Pb, and 6.1 (mg·kg^−1^·d^−1^) for Cd [42]. The remaining parameters are consistent with those used in Equation (3).

### 2.6. Statistical Analysis

Analytical results below the LOD were reported as not detected and replaced with LOD/2 to facilitate subsequent data analysis [43]. Statistical analysis was performed using Microsoft Excel 2021. Descriptive statistics for As, Hg, Pb, Cd, and Cu concentrations were generated across different sample categories. Since most of the data did not have a normal distribution (*p* < 0.05, Shapiro–Wilk test), the non-parametric Kruskal–Wallis test and the post hoc test for pairwise comparisons were used to examine the differences between groups for each metal. Significant values were adjusted by the Bonferroni correction for multiple testing. SPSS (Version 27.0) tests whether data are suitable for PCA. PCA and HCA were used to elucidate the differences and potential sources of PTE contamination among different medicinal parts by using Origin 2021. Furthermore, the pollution indices (*P_i_* and *P_N_*) and health risk assessment values (*THQ* and *CR*) were calculated according to established formulas. To quantify the uncertainties in the health risk assessment, this study employed the Oracle Crystal Ball add-in (Version 11.1.3) to conduct a probabilistic risk assessment within the Microsoft Excel environment.

## 3. Results

### 3.1. Descriptive Statistics of HM Concentrations in TCMMs

The concentrations, detection rates, and exceedance rates of five PTEs in TCMMs are shown in Table 1 and Table S5. The overall detection rate of TCMMs was 63.0%, and the detection rate of five PTEs in three types of medicinal parts was further calculated. As and Cu were detected in over 80% of root and rhizome samples. In samples of stems and leaves, whole herbs, flowers, fruits and seeds, the detection rates for As, Cu, and Hg exceeded 70%. In animal-derived decoction pieces, the detection rates of all PTEs exceeded 90%; in particular, As and Cu were detected in 100% of the samples. In these TCMMs, the relatively high detection rates of PTEs suggest that these five elements are widely present in such materials. The overall exceedance rates of As, Hg, and Cu across the 59 types of decoction pieces were all >7%, but the differences between various categories were not reflected. Therefore, exceedance rates were further calculated for three different types and medicinal parts. In root and rhizome samples, exceedance was primarily observed for As and Cu, in the decreasing order of Cu = As > Hg = Cd > Pb. Notably, no exceedance of Pb was observed in this type. In aboveground parts, exceedance was primarily observed for Hg and Cu, following the order Hg > Cu > Pb > As = Cd, with no exceedances for As and Cd. In animal-derived decoction pieces, exceedance was observed for all PTEs, in the order As > Pb = Cu > Hg > Cd. These results show that different medicinal parts exhibited distinct accumulation capacities for these PTEs. To quantify these differences, we analyzed the concentrations of each element across the three categories of medicinal materials. The Kruskal–Wallis test revealed a significant overall difference among the three groups (*p* < 0.05), shown in Table S4. The results show that the elements As, Hg, and Cu exhibit significant differences in animal-derived decoction pieces and root and rhizome samples. As, Pb, and Cd show significant differences in enrichment between animal-derived decoction pieces and aboveground parts. The enrichment of Cd differs markedly between underground parts and aboveground medicinal materials.

**Figure 2:**
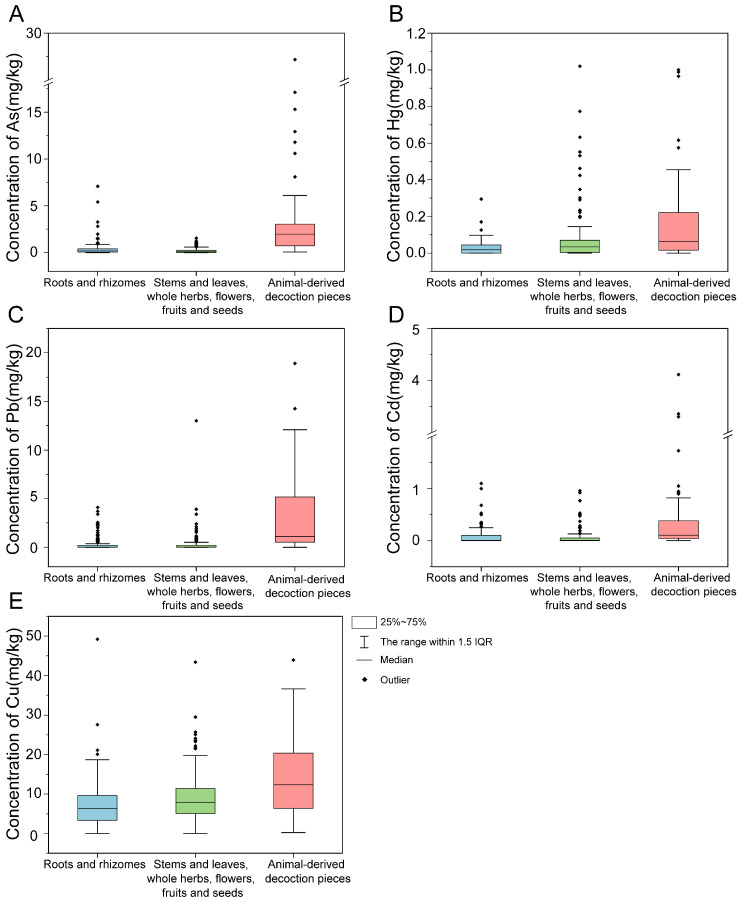
PTE concentrations in roots and rhizomes; stems and leaves, whole herbs, flowers, fruits and seeds; and animal-derived decoction pieces: (**A**) As; (**B**) Hg; (**C**) Pb; (**D**) Cd; (**E**) Cu.

The concentration ranges of As, Hg, Pb, Cd, and Cu across the 422 batches of TCMMs were 0–27.2, 0–1.02, 0–55.0, 0–4.11, and 0–49.20 mg·kg^−1^, respectively. Although the maximum concentrations significantly exceeded limits, the average concentrations were below the standard limits, indicating that extreme values occurred in certain TCMMs. Subsequent analysis of PTE concentrations across different types and medicinal parts revealed that, in root and rhizome samples—As: 0–7.09, Hg: 0–0.29, Pb: 0–4.10, Cd: 0–1.10, Cu: 0–49.20 mg·kg^−1^; in aboveground parts—As: 0–1.54, Hg: 0–1.02, Pb: 0–13.0, Cd: 0–0.96, Cu: 0–43.4 mg·kg^−1^; and in animal-derived decoction pieces—As: 0.07–27.18, Hg: 0–1.00, Pb: 0–55.0, Cd: 0–4.11, Cu: 0.23–43.9 mg·kg^−1^. These findings indicated a significant span in the concentration ranges of PTEs. Box plots of the five PTEs across the three types of medicinal decoction pieces further revealed their distribution characteristics (Figure 2). The results showed that As had a highly dispersed distribution in root and rhizome samples. Hg exhibited substantial variability in aboveground parts. In animal-derived decoction pieces, As, Pb, and Cd displayed pronounced within-group differences. Notably, Cu showed considerable variation across all categories.

### 3.2. PCA and Cluster Analysis

The Kaiser–Meyer–Olkin test and Bartlett’s test of sphericity were conducted using SPSS (27.0). The KMO value (0.515) exceeded the acceptable threshold of 0.5, and Bartlett’s test was statistically significant (*p* < 0.001), indicating that the data was appropriate for PCA. In addition, HCA was employed to assess the similarity between PTEs, thereby further validating the PCA results.

**Figure 3:**
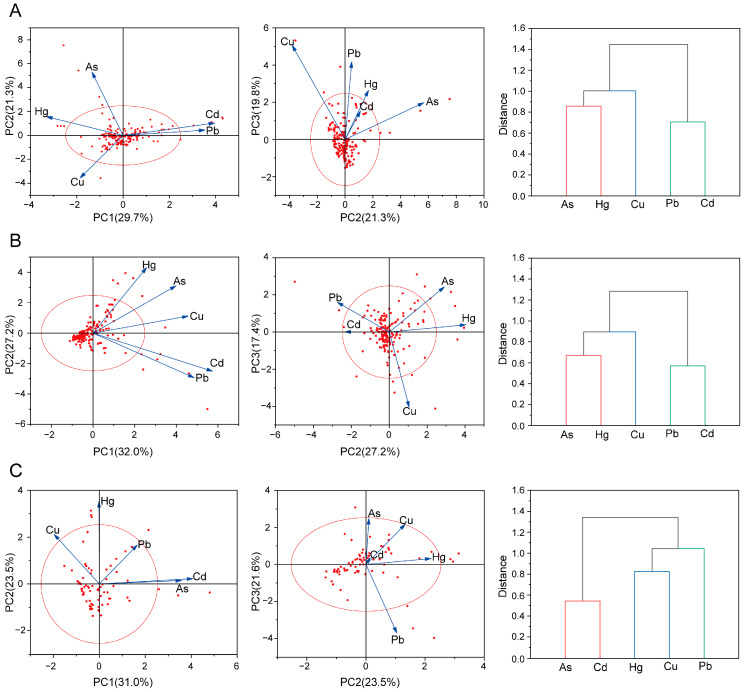
PCA and cluster analysis: (**A**) Roots and rhizomes; (**B**) Stems and leaves, whole herbs, flowers, fruits and seeds; (**C**) Animal-derived decoction pieces. Red circles indicate the 95% confidence intervals in PCA plots. Each red dot represents one sample.

The PCA results for root and rhizome samples are presented in Figure 3A. Three principal components were extracted, collectively explaining 70.8% of the total variance. PC1 (29.7%) showed strong positive loadings on Pb and Cd, indicating potential common sources. PC2 (21.3%) was primarily influenced by As and Hg, while PC3 (19.8%) was mainly characterized by Cu. Cluster analysis grouped the five PTEs into three distinct clusters: (1) As and Hg, (2) Cu, and (3) Pb and Cd. The results of the clustering analysis are consistent with those revealed by PCA. A similar pattern was observed for aboveground parts (Figure 3B, 76.6% variance explained), where PC1 was dominated by Pb and Cd, PC2 by As and Hg, and PC3 by Cu; clustering again yielded the same three groups. In contrast, animal-derived decoction pieces (Figure 3C, 76.1%) showed distinct profiles, with PC1 driven by As and Cd, PC2 by Hg and Cu, and PC3 by Pb, which also aligned with the clustering results.

For plant-derived materials, PCA and HCA consistently grouped the five PTEs into three clusters (As–Hg, Cu, Pb–Cd), likely reflecting common patterns of uptake and accumulation. In contrast, animal-derived decoction pieces showed a distinct grouping (As–Cd, Hg–Cu, Pb), highlighting fundamental biological differences between plants and animals in metal uptake, metabolism, and bioaccumulation.

### 3.3. Results of Evaluation of PTEs Pollution Index

**Figure 4:**
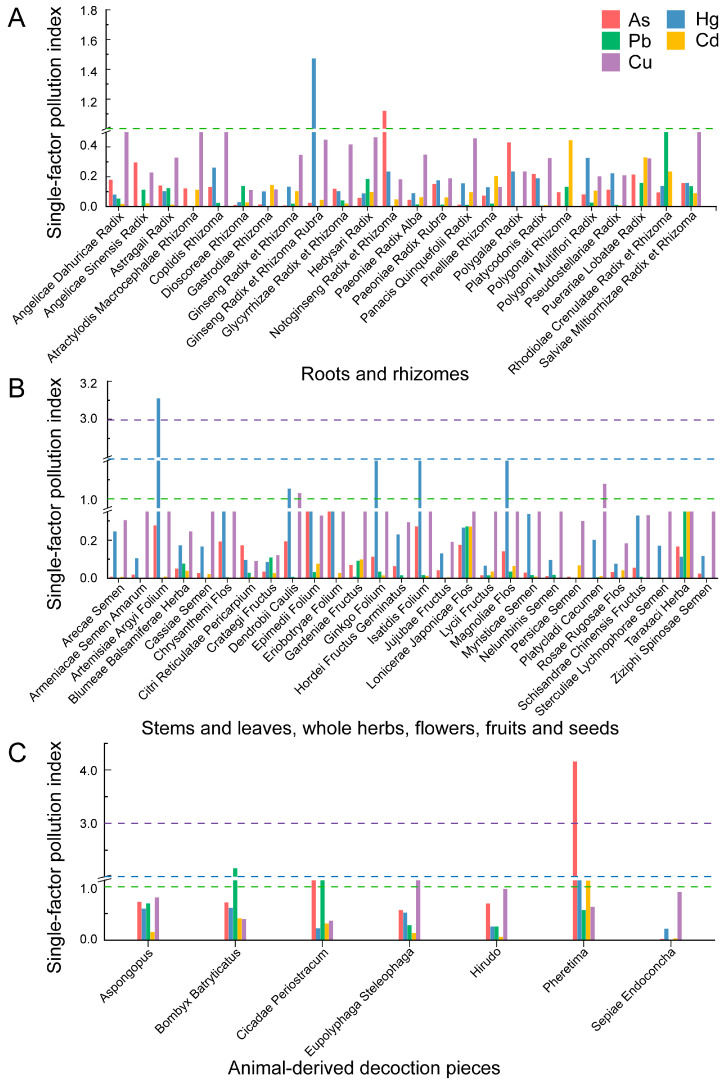
Single-factor pollution index (*P_i_*) plots: (**A**) Roots and rhizomes; (**B**) Stems and leaves, whole herbs, flowers, fruits and seeds; (**C**) Animal-derived decoction pieces.

**Figure 5:**
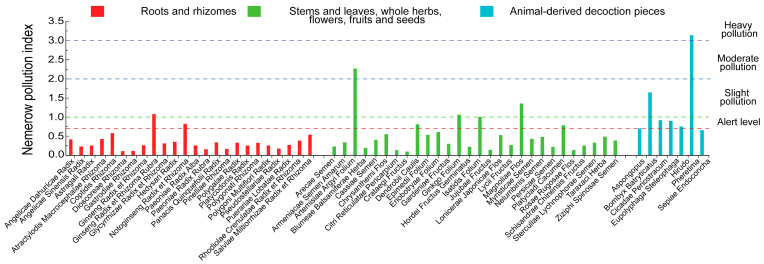
Nemerow Pollution Index (*P_N_*) plots. The red dashed line indicates *P_N_* = 0.7; the green dashed line indicates *P_N_* = 1; the blue dashed line indicates *P_N_* = 2; and the purple dashed line indicates *P_N_* = 3.

The results of the *P_i_* and *P_N_* are shown in Figure 4 and Figure 5. Among the 422 collected batches of medicinal materials, 16 were found to be contaminated based on the single-factor pollution index (*P_i_* > 1). Specifically, there were 2 cases of heavy pollution (*P_i_* > 3), 1 case of moderate pollution (2 < *P_i_* ≤ 3), and 13 cases of slight pollution (1 < *P_i_* ≤ 2). The major PTEs contributing to contamination are shown in Figure 4 and detailed in Tables S6 and S7. In the roots and rhizomes, slight pollution was observed in *Notoginseng Radix et Rhizoma* (As, 1.12) and *Ginseng Radix et Rhizoma Rubra* (Hg, 1.48). Among stems and leaves, whole herbs, flowers, fruits and seeds, one sample of *Artemisiae Argyi Folium* exhibited heavy pollution (Hg, 3.11), while six samples showed slight pollution: *Platycladi Cacumen* (Cu, 1.0788), *Isatidis Folium* (Hg, 1.33), *Ginkgo Folium* (Hg, 1.44), *Dendrobii Caulis* (Hg, 1.05; Cu, 1.03), and *Magnoliae Flos* (Hg, 1.83). In animal-derived decoction pieces, *Pheretima* showed heavy pollution (As, 4.16), *Bombyx Batryticatus* exhibited moderate contamination (Pb, 2.16), and five samples showed slight pollution: *Cicadae Periostracum* (As, 1.13; Pb, 1.14), *Pheretima* (Hg, 1.23; Cd, 1.12), and *Eupolyphaga Steleophaga* (Cu, 1.15). Further analysis revealed that Hg levels in *Artemisiae Argyi Folium* exceeded the regulatory limit by 2 to 3.8 times. As levels in *Pheretima* generally surpassed the permissible limit, with one batch reaching 14 times the standard. Approximately one-third of *Bombyx Batryticatus* batches exceeded the limit for Pb, with three samples showing particularly high concentrations. These findings highlight severe Hg contamination in *Artemisiae Argyi Folium* and As contamination in *Pheretima*, necessitating further attention to the health risks posed to humans by these medicinal materials. Moreover, the elevated Pb levels in *Bombyx Batryticatus* suggest that regional environmental pollution at the production site may be a contributing factor, warranting closer monitoring of geographical origins.

The *P_i_* revealed that multiple PTEs were present in *Dendrobii Caulis*, *Cicadae Periostracum*, and *Pheretima*. However, as the single-factor index only reflects contamination from individual elements, it cannot quantify the cumulative risk posed by the combined effects of multiple PTEs. Therefore, the *P_N_* was employed to comprehensively evaluate the synergistic effects of five PTEs in medicinal materials. The results are presented in Figure 5 and Tables S6 and S8. In total, 14 medicinal materials exceeded the alert level: 1 exhibited heavy pollution (*P_N_* > 3), 1 exhibited moderate pollution (2 < *P_N_* ≤ 3), 5 exhibited slight pollution (1 < *P_N_* ≤ 2), and 7 were within the alert level (0.7 < *P_N_* ≤ 1). Among roots and rhizomes, *Ginseng Radix et Rhizoma Rubra* showed slight pollution, mainly attributed to Hg, while *Notoginseng Radix et Rhizoma* reached the alert level, with As being the main pollutant. All other samples in this group had *P_N_* < 0.7, indicating safe levels. In the samples of stems and leaves, whole herbs, flowers, fruits and seeds, *Artemisiae Argyi Folium* exhibited moderate pollution. *Isatidis Folium*, *Ginkgo folium*, and *Magnoliae Flos* showed slight pollution, while *Platycladi Cacumen* and *Dendrobii Caulis* fell within the alert level. The main pollutants in this group were Hg and Cu. For animal-derived decoction pieces, all samples except *Sepiae Endoconcha* had *P_N_* > 0.7. *Pheretima* was classified as heavy pollution, *Bombyx Batryticatus* as slight pollution, and *Aspongopus*, *Hirudo*, *Eupolyphaga Steleophaga*, and *Cicadae Periostracum* fell within the alert level. All five PTEs were implicated across these samples. The results suggest that plant-derived medicinal materials were mainly contaminated by As, Hg, and Cu, whereas animal-derived products displayed more complex contamination patterns involving all five PTEs. The main pollutants differed significantly among animal medicines (e.g., As in *Pheretima*, Pb in *Bombyx Batryticatus,* and As/Pb in *Cicadae Periostracum*), likely due to differences in ecological behavior, dietary sources, and metabolic pathways related to PTE accumulation. The specific mechanism remains to be elucidated through further research.

### 3.4. Health Risk Assessment

**Figure 6:**
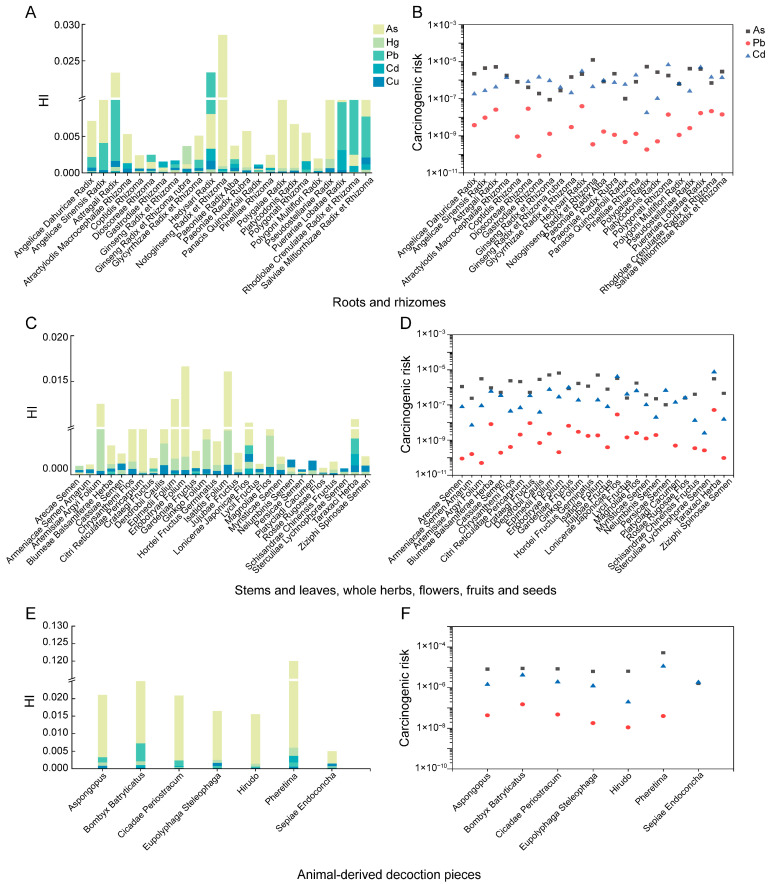
Non-carcinogenic risk (**A**,**C**,**E**) and carcinogenic risk (**B**,**D**,**F**) assessment for roots and rhizomes; stems and leaves, whole herbs, flowers, fruits and seeds; and animal-derived decoction pieces.

This study employed *THQ* and *CR* assessment methods to evaluate the non-carcinogenic and carcinogenic health risks associated with five PTEs in TCMMs. The results for the 422 batches of 59 types of medicinal materials are presented in Figure 6, Tables S9-1–S10-3. The *THQ* and *HI* values of PTEs in TCMMs were both <1, suggesting that there were no non-carcinogenic health risks for adults. Among the different types of medicinal materials, the risk levels follow this order: animal-derived decoction pieces > roots and rhizomes > stems and leaves, whole herbs, flowers, fruits and seeds. In the animal-derived groups, the highest *HI* was observed in *Pheretima* (1.20 × 10^−1^), primarily influenced by As. In the roots and rhizomes, *Notoginseng Radix et Rhizoma* exhibited the highest *HI* (2.85 × 10^−2^), mainly attributed to As exposure. Among stems and leaves, whole herbs, flowers, fruits and seeds, *Eriobotryae Folium* had the highest *HI* (1.66 × 10^−2^), primarily due to Hg.

The results of the carcinogenic risk analysis (Figure 6, Table S7) showed that the *CR* values for all TCMMs were <1 × 10^−4^. Among the three carcinogenic PTEs (As, Cd, and Pb), the ranking in all samples was As > Cd > Pb. Importantly, this hierarchy remained consistent within underground parts, aboveground parts and animal-derived decoction pieces, indicating a consistent trend in carcinogenic risk distribution across different types of TCMMs. In roots and rhizomes, the highest carcinogenic risks were associated with specific elements in the following medicinal materials: As in *Notoginseng Radix et Rhizoma* (1.24 × 10^−5^), Pb in *Hedysari Radix* (3.91 × 10^−8^), and Cd in *Polygonati Rhizoma* (6.71 × 10^−6^). For stems and leaves, whole herbs, flowers, fruits and seeds, the highest risks were observed for As in *Eriobotryae Folium* (6.59 × 10^−6^), Pb (5.10 × 10^−8^) and Cd in *Taraxaci Herba* (7.47 × 10^−6^). Among animal-derived decoction pieces, the most significant carcinogenic risks were found in As (5.13 × 10^−5^) and Cd (1.12 × 10^−5^) in *Pheretima*, and Pb (1.51 × 10^−7^) in *Bombyx Batryticatus.* While the health risks associated with PTEs in three types of TCMMs are relatively low, the potential hazards of specific PTEs in certain materials remain a concern.

### 3.5. Health Risk Assessment Based on Monte Carlo Simulation

Using a Monte Carlo simulation approach, 10,000 random iterations were conducted to estimate the *HI* and *CR* values for the three types of TCMMs. Consult references to define the data ranges for relevant parameters (PTEs concentrations, IRD, EF, BW) [44], shown in Table S11. As summarized in the table, the *HI* values for all three categories were <1, and the probability of *HI* > 1 was 0%, indicating the absence of non-carcinogenic risks under the parameter ranges specified in this study (Table S12). For carcinogenic risk, the 95th percentile values of the *CR* for all three categories were <1 × 10^−4^. The probabilities of the total *CR* > 1 × 10^−6^ for the three categories were 53.78%, 33.57% and 99.18%, respectively, with the aboveground parts showing a 0.05% probability of >1 × 10^−4^. These findings indicate that the carcinogenic risks posed by these medicinal materials are of notable concern. Following the simulations, the sensitivity analysis function in Crystal Ball was used to quantify the contribution of each input variable to the uncertainty in the *HI* and *CR* estimates (Table 2). The results showed that metal concentrations and daily intake were identified as the primary contributors to uncertainty in the risk assessment.

## 4. Discussion

TCMMs are widely used worldwide, but the permissible limits for PTEs differ among national pharmacopoeias. According to the International Organization for Standardization, the limits for PTEs in medicinal materials are Pb ≤ 10, Cd ≤ 2, As ≤ 4, and Hg ≤ 3 mg·kg^−1^. The World Health Organization recommends maximum permissible levels of Pb ≤ 10.0 mg·kg^−1^ and Cd ≤ 0.3 mg·kg^−1^ in medicinal plant materials. The United States Pharmacopeia defines limits for elemental impurities in botanical drugs as Pb ≤ 5, Cd ≤ 0.5, As ≤ 2, total Hg ≤ 1, and MeHg ≤ 0.2 mg·kg^−1^. The European Pharmacopoeia specifies the following limits for herbal drugs and preparations: Pb ≤ 5, Cd ≤ 1, and Hg ≤ 0.1 mg·kg^−1^ [45,46]. These standards primarily focus on plant-based medicinal materials, with relatively limited attention to animal-derived products. The Chinese Pharmacopoeia attempts to cover a broader range, with unified limits for PTEs in plant-based drugs and decoction pieces: Pb ≤ 5, Cd ≤ 1, As ≤ 2, Hg ≤ 0.2, and Cu ≤ 20 mg·kg^−1^ [6]. For animal-derived decoction pieces, species-specific limits have been established for individual PTEs. For example, the permissible limits for *Hirudo* are Pb ≤ 10, Cd ≤ 1, Hg ≤ 1, and As ≤ 5 mg·kg^−1^. However, certain commonly used animal decoction pieces, such as *Cicadae Periostracum* and *Pheretima*, still lack established regulatory standards. Notably, a comparison of international standards indicates that the PTEs limits set by the Chinese Pharmacopoeia are not less stringent than those in other pharmacopoeias, and are even stricter for elements such as lead and arsenic.

The accumulation of PTEs not only compromises the safety of TCMMs but may also impair their therapeutic efficacy [47]. Based on the non-parametric analysis of PTE concentrations across the different types of TCMMs, in combination with the findings on exceedance rates, we found that the PTE profiles differed among the various medicinal parts. In roots and rhizomes, exceedances were primarily concentrated in As and Cu, consistent with previous reports of high levels of Cd, Pb, Hg, and particularly As in *Astragali Radix* from Heilongjiang [48]. A previous study on rhizome-type medicinal materials also found high Cu levels, which is consistent with our results. However, that study reported Cd exceedance, while the main excessive elements in our study were different [33]. This difference may be related to the scope of sampling. The previous study examined only four rhizome species, whereas our study included more types of medicinal materials, allowing us to better reflect their varied abilities to accumulate different elements. For stems and leaves, whole herbs, flowers, fruits and seeds, contamination was associated not only with As and Cu but also with Hg. Research has reported that the significant accumulation of Hg occurred in leaves, a finding consistent with our results [49]. Previous reviews have also highlighted As, Hg, and Pb as key contaminants of concern in these categories of medicinal materials [50,51]. Our results further confirm the importance of As and Hg, suggesting that their contamination risks are common and persistent, likely due to their high environmental mobility and plant uptake and transport characteristics. Unlike earlier reviews, Pb did not show clear enrichment in our samples, whereas Cu had a more pronounced influence. This difference may be related to environmental conditions in the production areas—such as soil composition, farming practices, and local industrial pollution—which can alter how different elements accumulate in plants. Animal-derived decoction pieces showed more serious PTE contamination, and their PTEs levels differed significantly from those in underground parts and aboveground parts. This pattern may be driven by amplified bioaccumulation, worsening environmental contamination (especially in aquatic systems), potential pollution during farming, and possible secondary contamination during processing and storage. When compared with findings from other countries, the levels of PTEs contamination observed in our study are generally consistent with previous reports. For example, in traditional herbal products commonly used in Pakistan’s Khyber Pakhtunkhwa region, all samples contained toxic heavy metals, with As showing particularly severe contamination and posing clear health risks [52]. Similarly, in the Qassim region of central Saudi Arabia, all 32 commercially available herbal samples tested positive for heavy metals [53].

Previous studies have demonstrated that PTEs with high loadings on the same principal component usually share similar sources [54]. In our study, PCA and HCA revealed consistent clustering patterns in plant-derived materials (Pb-Cd, As-Hg, Cu), while animal-derived decoction pieces showed distinct groupings (As-Cd, Hg-Cu, Pb), indicating different enrichment characteristics. Cd and Pb contamination is largely linked to industrial activities (e.g., battery production, smelting, electroplating, coal combustion) as well as agricultural inputs such as fertilizers and livestock manure [55,56,57]. As and Hg are characterized by high toxicity, persistence, and bioaccumulative potential. Their environmental pollution is mainly originated from coal combustion, metal smelting, and the historical use of pesticides and fertilizers [58]. Cu inputs are primarily associated with fertilizers, livestock manure, and irrigation water [59]. Pb and Cd, strongly adsorbed by soils, tend to accumulate in roots and rhizomes through soil–root transfer, whereas As and Hg, being more mobile, also enter aboveground tissues via atmospheric deposition. The divergent patterns observed in animal-derived materials likely reflect species-specific metabolic pathways and regulatory mechanisms governing HM uptake, transport, and storage.

The assessment of PTE contamination in medicinal materials using the single factor index and the Nemerow pollution index revealed that 14 of the 59 medicinal materials exceeded the threshold (*P_N_* > 1). *Notoginseng Radix et Rhizoma* and *Ginseng Radix et Rhizoma Rubra* (roots and rhizomes type) were primarily contaminated with As and Hg. In the stems and leaves, whole herbs, flowers, fruits and seeds category, *Artemisiae Argyi Folium*, *Platycladi Cacumen*, *Isatidis Folium*, *Ginkgo folium*, *Dendrobii Caulis*, and *Magnoliae Flos* were contaminated with Hg and Cu. Among animal-derived decoction pieces, all samples except *Sepiae Endoconcha* were affected by various PTEs. The results demonstrate that different medicinal parts are characterized by distinct dominant pollution elements. To evaluate the safety of PTEs in TCMMs, this study employed *THQ* and *HI* for non-carcinogenic risk assessment, and *CR* for evaluating potential cancer risk. The results showed that the *THQ* values of As, Hg, Pb, Cd, and Cu, as well as the *HI* values, were all <1; the Monte Carlo simulation results also show that the probability of *HI* being >1 is 0%. Assuming that all species of As, Pb, and Cd in TCMMs exist in their most toxic forms, the *CR* values of Pb for all samples were <1 × 10^−6^, while the *CR* values of As and Cd were <1 × 10^−4^. Monte Carlo simulation further showed that the daily intake and metal concentrations were the main sources of uncertainty, with As being the dominant contributor to carcinogenic risk. This pattern is consistent with findings by Gholamreza Ebrahimzadeh et al., who reported that THQ and CR were largely driven by intake levels, metal concentrations, and body weight [60].

In this study, we observed inconsistencies between pollution indices and health risk assessment results. For instance, according to both the single-factor pollution index and the Nemerow pollution index, *Notoginseng Radix et Rhizoma* in the roots and rhizomes exhibited severe contamination. Consistently, the health risk assessment also identified *Notoginseng Radix et Rhizoma* (2.85 × 10^−2^) as having the highest non-carcinogenic risk, with As being the major contributing element. The highest carcinogenic risks were associated with *Notoginseng Radix et Rhizoma* (As), *Hedysari Radix* (Pb), and *Polygonati Rhizoma* (Cd). Similarly, among stems, leaves, whole herbs, flowers, fruits and seeds, *Artemisiae Argyi Folium* was heavily and moderately contaminated, mainly due to the influence of Hg, but in the risk assessment, *Eriobotryae Folium* had a higher health risk. We further examined the factors responsible for these discrepancies. The pollution indices are designed to determine whether the PTE concentrations in medicinal materials exceed regulatory limits, and their results depend mainly on the comparison between measured levels and standard thresholds [61]. As a result, *Notoginseng Radix et Rhizoma* shows a high pollution level due to elevated As, and *Artemisiae Argyi Folium* appears heavily contaminated because of marked Hg enrichment. In contrast, health risk assessment focuses on the potential health hazards after long-term human intake. Its result is determined by “pollutant content—toxicity intensity—exposure dose”, so the ranking may not be consistent with the pollution index. The factors that determine health risk are mainly as follows: (1) Variations in toxicity weighting [62]. Although Hg is highly enriched in *Artemisiae Argyi Folium*, its carcinogenic and non-carcinogenic toxicity weights are relatively low. Therefore, even at high concentrations, its contribution to overall health risk remains limited. In comparison, As and Cd have higher carcinogenic slope factors, meaning that even lower concentrations can lead to considerable carcinogenic risk. Consequently, *Artemisiae Argyi Folium* shows a high pollution index due to a high Hg content, whereas *Eriobotryae Folium* poses a higher health risk because it contains the highest As level and has stronger toxicity. (2) Effects of exposure pathways and intake levels [63]. Among root and rhizome materials, *Hedysari Radix* has the highest daily intake, while *Polygonati Rhizoma*, in addition to its high intake, also has a large Cd carcinogenic slope factor. Therefore, the combination of high intake and highly toxic elements leads to elevated carcinogenic risk in these materials. Overall, high pollution does not necessarily equate to high health risk. Health risk is closely linked to metal toxicity (*RfD*, *CSF*), exposure pathways, and intake levels—factors that are not captured by pollution indices.

## 5. Conclusions

This study evaluated five PTEs (As, Hg, Pb, Cd, and Cu) in 59 species and 422 batches of TCMMs from roots and rhizomes, stems and leaves, whole herbs, flowers, fruits and seeds, and animal-derived decoction pieces. The main findings are summarized as follows:

(1) Contamination profiles differed by material type and medicinal part, with animal-derived decoction pieces showing notably higher metal loads. It is recommended that corresponding limit standards be established for all other single-ingredient animal-derived medicinal materials, excluding *Sepiae Endoconcha* and *Hirudo*.
(2) Multivariate analyses revealed distinct source patterns: underground and aboveground parts shared similar metal signatures, whereas animal-derived decoction pieces exhibited clearly different profiles.
(3) Key pollutants varied across medicinal parts: As and Hg were more relevant for underground parts, Hg and Cu for aboveground parts, while all five elements required attention in animal-derived decoction pieces.
(4) Health risk assessment indicated generally low risks: The *HI* values of underground parts, aboveground parts, and animal-derived decoction pieces fell within the ranges of (1.13 × 10^−3^~2.85 × 10^−2^), (5.86 × 10^−4^~1.66 × 10^−2^), (5.01 × 10^−3^~1.20 × 10^−1^), respectively, and all *CR* values were <1 × 10^−4^. Sensitivity analysis showed that metal concentrations and daily intake were the main contributors to uncertainty. These findings highlight the importance of controlling metal contamination in raw materials and standardizing dosage to further reduce potential health risks.

## Figures and Tables

**Table 1:** Overall and classified comparison of PTE concentration distribution and contamination rates in TCMMs.

PTEs	Statistical Indicators	Overall	Roots and Rhizomes	Stems and Leaves, Whole Herbs, Flowers, Fruits and Seeds	Animal-Derived Decoction Pieces
As	range (mg·kg^−1^)	0–27.2	0–7.09	0–1.54	0.07–27.18
	mean ± sd	0.78 ± 2.17	0.37 ± 0.77 **	0.21 ± 0.31 ***	3.03 ± 4.35
	detection rate (%)	82.70%	84.20%	73.50%	100.00%
	exceedance rate (%)	8.30%	2.30%	0.00%	41.30%
Hg	range (mg·kg^−1^)	0–1.02	0–0.29	0–1.02	0–1.00
	mean ± sd	0.067 ± 0.136	0.03 ± 0.05 **	0.07 ± 0.14	0.15 ± 0.22
	detection rate (%)	72.00%	57.60%	76.50%	96.00%
	exceedance rate (%)	8.10%	1.70%	8.80%	24.00%
Pb	range (mg·kg^−1^)	0–55.0	0–4.10	0–13.0	0–55.0
	mean ± sd	1.06 ± 4.19	0.33 ± 0.71	0.33 ± 1.13 **	4.45 ± 9.03
	detection rate (%)	63.50%	55.90%	58.20%	93.30%
	exceedance rate (%)	4.70%	0.00%	0.60%	25.30%
Cd	range (mg·kg^−1^)	0–4.11	0–1.10	0–0.96	0–4.11
	mean ± sd	0.125 ± 0.360	0.08 ± 0.16 ***	0.06 ± 0.15 #	0.38 ± 0.74
	detection rate (%)	63.00%	55.90%	56.50%	94.70%
	exceedance rate (%)	1.40%	1.70%	0.00%	6.70%
Cu	range(mg·kg^−1^)	0–49.20	0–49.20	0–43.4	0.23–43.9
	mean ± sd	8.98 ± 7.20	7.06 ± 5.86 **	8.72 ± 6.49	14.1 ± 9.04
	detection rate (%)	90.50%	84.80%	92.40%	100.00%
	exceedance rate (%)	7.80%	2.30%	5.90%	25.30%

Animal-based decoction pieces vs. roots and rhizomes or stems and leaves, whole herbs, flowers, fruits and seeds (**, *p* < 0.01; ***, *p* < 0.001); #, roots and rhizomes vs. stems and leaves, whole herbs, flowers, fruits and seeds (#, *p* < 0.05).

**Table 2:** Summary of the sensitivity analysis results.

Parameters	Roots and Rhizomes-HI Contribution %	Stems and Leaves, Whole Herbs, Flowers, Fruits and Seeds-HI Contribution %	Animal-Derived Decoction Pieces-HI Contribution %	Roots and Rhizomes-CR Contribution %	Stems and Leaves, Whole Herbs, Flowers, Fruits and Seeds-CR Contribution %	Animal-Derived Decoction Pieces-CR Contribution %
As	61.0%	69.4%	60.7%	48.2%	72.3%	51.8%
Hg	2.5%	10.4%	0.1%	0.0%	0.0%	0.0%
Pb	1.2%	0.5%	0.1%	0.0%	0.0%	0.0%
Cd	0.3%	0.5%	0.0%	19.3%	18.8%	2.7%
Cu	0.3%	1.2%	0.0%	0.0%	0.0%	0.0%
IR	23.6%	7.9%	20.7%	21.6%	3.8%	24.1%
EF	9.5%	8.5%	15.4%	9.3%	4.2%	18.1%
BW	1.7%	1.5%	3.0%	1.6%	0.9%	3.4%

## Data Availability

The original contributions presented in this study are included in the article/Supplementary Material. Further inquiries can be directed to the corresponding author.

## Supplementary Materials

The following supporting information can be downloaded at: https://www.mdpi.com/article/10.3390/toxics14010040/s1.
