# Supplementary material for: Potentially Toxic Elements Accumulation and Health Risk Evaluation in Different Parts of Traditional Chinese Medicinal Materials
Source: Toxics. 2025 Dec 30;14(1):40. doi: 10.3390/toxics14010040 (PMC12846019; doi:10.3390/toxics14010040)
Supplement: Supplementary file 1 [file toxics-14-00040-s001.zip › toxics-3976152-supplementary.pdf]

Supplementary Materials
for

Potentially Toxic Elements Accumulation and Health Risk Evaluation in Different Parts of Traditional Chinese medicinal materials

Jie Pan [1,2,†], Di Huang [1,2,3,†], Xue Ma [1,2], Di Zhu [a,b], Yuan Lu [1,2], Chunhua Liu [1,2], Lin Zheng [1,2], Yongjun Li [1,2,3,*], Jia Sun [1,2,*]

1 State Key Laboratory of Discovery and Utilization of Functional Components in Traditional Chinese Medicine, Engineering Research Center for the Development and Application of ethnic Medicine and TCM (Ministry of Education),Guizhou Provincial Engineering Research Center for the Development and Application of ethnic Medicine and TCM, Guizhou Medical University, Guian New Area 561113, China;

2 Guizhou Key Laboratory of Modern Traditional Chinese Medicine Creation, Guiyang, 550004, China;

3 School of Pharmacy, Guizhou Medical University, Guian New Area 561113, China

* Correspondence: sunjia@gmc.edu.cn (J. Sun), liyongjun026@126.com (Y. Li)

† These authors contributed equally to this work.

Table S1 *Instrumental parameter settings*

Pb, Cd Detection was performed using graphite furnace atomic absorption spectrophotometry (GFAAS), Cu Detection was performed using flame atomic absorption spectrophotometry (FAAS), As, Hg were detected by Atomic Fluorescence Spectrometry (AFS). Different metal ions require different operating parameters. See Tables S1-1, S1-2, and S1-3.

Table S1-1 *Graphite furnace atomic absorption spectrometry instrument parameters (Pb, Cd)*

PTEs	Spectrum（nm）	Slit Width（nm）	Lamp Current(mA)	Drying Temp.(℃)	Drying Time(s)	Ashing Temp.(℃)	Ash Time(s)	Atomization Temp.(℃)	Atomization Time(s)	Matrix Modifier
Pb	238.3	0.7	60	110	30	950	20	1600	5	$Mg(NO_3)_2$
Cd	228.8	0.7	60	110	25	800	25	1700	5	$Mg(NO_3)_2$

Table S1-2 *Flame atomic absorption spectrometry instrument parameters (Cu)*

PTEs	Wavelength（nm）	Slit Width（nm）	Lamp Current (mA)	Acetylene Flow (L/min)	Air Flow (L/min)
Cu	324.7	1.4	4	1.6	6

Table S1-2 *Atomic Fluorescence Spectrometry instrument parameters (Hg, As)*

PTEs	Wavelength（nm）	Atomization Temp.（℃）	Lamp Current(mA)	Carrier Gas Flow（L/min）	Sheath Gas Flow（L/min）	Reducing Agent	Carrier Solution
Hg	253.6	800	80	400	1	1%$NaBH_4$, 0.3%NaOH	0.5%HCl
As	193.7	800	80	400	1		

Table S2-1 *Calibration curves and correlation coefficient for 5 PTEs to be determined*

PTEs	Regression Eq. & Corr. Coefficient (R)	R-value	Linear Range
As	y=0.01048x＋0.00348	0.999 66	1 ~ 20

Hg	y=0.00732x−0.00062	0.999 85	0.5 ~ 10
Pb	y=0.00153x−0.00085	0.998 01	10 ~ 50
Cd	y=0.03977x+0.00163	0.998 19	1 ~ 5
Cu	y=0.15982x+0.00074	0.999 93	100 ~ 1000

Table S2-2 *The precision test results of 5PTEs (IF)*

PTEs	Pb	Cd	Cu	As	Hg
1	0.0650	0.0138	0.0593	1369	1249
2	0.0640	0.0148	0.0593	1356	1213
3	0.0650	0.0148	0.0593	1363	1218
4	0.0680	0.0154	0.0593	1357	1212
5	0.0640	0.0159	0.0606	1359	1215
6	0.0600	0.0157	0.0598	1328	1290
mean	0.0640	0.0151	0.0596	1355	1233
RSD%	4.2	5.1	0.80	1.0	2.6

Table S2-3 *The repeatability test results of 5PTEs* (mg/kg)

PTEs	As	Hg	Cu	Pb	Cd
1	0.420	0.129	12.4	1.70	0.163
2	0.433	0.113	13.5	1.73	0.169
3	0.441	0.130	12.6	1.92	0.167
4	0.454	0.113	13.4	1.59	0.171
5	0.453	0.112	15.1	1.66	0.174
6	0.456	0.125	14.1	1.75	0.173
mean	0.443	0.120	13.5	1.73	0.170
RSD%	3.3	7.2	7.2	6.6	2.4

Table S3-1 *The recovery test results of As*

Number	weighing the sample (g)	Content (μg)	Added amount (μg)	Measured quantity (μg)	Recovery rate%	Average recovery rate %	RSD %
1	0.2517	0.111	0.09	0.191	88.97	103.3	12
	0.2509	0.111	0.09	0.208	107.6		
	0.2500	0.111	0.09	0.213	113.3		
2	0.2509	0.111	0.10	0.200	89.21	99.10	9.5
	0.2505	0.111	0.10	0.219	108.0		
	0.2511	0.111	0.10	0.211	100.1		
3	0.2507	0.111	0.13	0.232	93.17	99.06	5.8
	0.2501	0.111	0.13	0.240	99.29		

	0.2502	0.111	0.13	0.247	104.7		

Table S3-2 *The recovery test results of Hg*

Number	weighing the sample (g)	Content (μg)	Added amount (μg)	Measured quantity (μg)	Recovery rate%	Average recovery rate %	RSD %
	0.2517	0.0300	0.02	0.0483	91.26		
1	0.2509	0.0301	0.02	0.0519	108.9	104.2	11
	0.2500	0.0301	0.02	0.0526	112.6		
	0.2509	0.0300	0.03	0.0640	113.3		
2	0.2505	0.0301	0.03	0.0576	91.80	105.3	11
	0.2511	0.0300	0.03	0.0633	110.8		
	0.2507	0.0300	0.04	0.0715	103.7		
3	0.2501	0.0300	0.04	0.0659	89.63	95.4	7.7
	0.2502	0.0300	0.04	0.0672	92.94		

Table S3-3 *The recovery test results of Pb*

Number	weighing the sample (g)	Content (μg)	Added amount (μg)	Measured quantity (μg)	Recovery rate%	Average recovery rate %	RSD %
	0.2501	0.432	0.2	0.645	106.6		
1	0.2513	0.432	0.2	0.606	87.19	99.00	11
	0.2507	0.432	0.2	0.638	103.2		
	0.2510	0.432	0.4	0.908	119.0		
2	0.2522	0.432	0.4	0.833	100.3	110.4	8.5
	0.2572	0.432	0.4	0.879	111.8		
	0.2509	0.432	0.6	1.08	107.9		
3	0.2511	0.432	0.6	1.18	124.5	113.5	8.4
	0.2517	0.432	0.6	1.08	108.1		

Table S3-4 *The recovery test results of Cd*

Number	weighing the sample (g)	Content (μg)	Added amount (μg)	Measured quantity (μg)	Recovery rate%	Average recovery rate %	RSD %
	0.2501	0.0425	0.03	0.0741	105.2		
1	0.2513	0.0425	0.03	0.0719	97.91	97.47	8.1
	0.2507	0.0425	0.03	0.0693	89.33		
	0.2510	0.0425	0.04	0.0810	96.28		
2	0.2522	0.0425	0.04	0.0832	101.8	103.3	7.7
	0.2572	0.0425	0.04	0.0873	111.9		
	0.2509	0.0425	0.05	0.0941	103.1		
3	0.2511	0.0425	0.05	0.0974	109.8	102.9	6.8

	0.2517	0.0425	0.05	0.0904	95.85		

Table S3-5 *The recovery test results of Cu*

Number	weighing the sample (g)	Content (μg)	Added amount (μg)	Measured quantity (μg)	Recovery rate%	Average recovery rate %	RSD %
	0.2501	3.38	2.5	5.83	97.67		
1	0.2513	3.38	2.5	6.27	115.6	105.3	8.8
	0.2507	3.38	2.5	5.95	102.6		
	0.2510	3.38	3.5	6.81	97.87		
2	0.2522	3.39	3.5	7.22	109.5	105.0	5.9
	0.2572	3.39	3.5	7.15	107.5		
	0.2509	3.38	4.0	7.31	98.14		
3	0.2511	3.38	4.0	7.50	102.9	102.4	4.0
	0.2517	3.38	4.0	7.64	106.3		

Table S4 *Number of Samples in 422 Batches of TCMMs*

Name	medicinal parts	number of batches	Source
Angelicae Dahuricae Radix	Roots and rhizomes	3	a, b
Angelicae Sinensis Radix	Roots and rhizomes	17	a, b
Astragali Radix	Roots and rhizomes	4	c
Atractylodis Macrocephalae Rhizoma	Roots and rhizomes	3	a, b
Coptidis Rhizoma	Roots and rhizomes	9	a, b
Dioscoreae Rhizoma	Roots and rhizomes	11	c
Gastrodiae Rhizoma	Roots and rhizomes	16	c
Ginseng Radix et Rhizoma	Roots and rhizomes	4	a, b, c
Ginseng Radix et Rhizoma Rubra	Roots and rhizomes	6	b, c
Glycyrrhizae Radix et Rhizoma	Roots and rhizomes	12	c
Hedysari Radix	Roots and rhizomes	3	a, b
Notoginseng Radix et Rhizoma	Roots and rhizomes	6	a, b, c
Paeoniae Radix Alba	Roots and rhizomes	4	c
Paeoniae Radix Rubra	Roots and rhizomes	5	a, b, c
Panacis Quinquefolii Radix	Roots and rhizomes	10	a, d
Pinelliae Rhizoma	Roots and rhizomes	9	a, b
Platycodonis Radix	Roots and rhizomes	4	b, c
Polygalae Radix	Roots and rhizomes	9	a, b, c
Polygonati Rhizoma	Roots and rhizomes	16	a, b, c
Polygoni Multiflori Radix	Roots and rhizomes	7	a, b
Pseudostellariae Radix	Roots and rhizomes	4	a, b
Puerariae Lobatae Radix	Roots and rhizomes	9	a, b, c

Rhodiolae Crenulatae Radix et Rhizoma	Roots and rhizomes	3	a, b, c
Salviae Miltiorrhizae Radix et Rhizoma	Roots and rhizomes	3	c
Artemisiae Argyi Folium	Stems, leaves and whole herbs	3	c
Blumeae Balsamiferae Herba	Stems, leaves and whole herbs	7	a, b
Dendrobii Caulis	Stems, leaves and whole herbs	4	b, c
Epimedii Folium	Stems, leaves and whole herbs	4	a, b, c
Eriobotryae Folium	Stems, leaves and whole herbs	4	c
Ginkgo Folium	Stems, leaves and whole herbs	4	c
Isatidis Folium	Stems, leaves and whole herbs	4	b, c
Platycladi Cacumen	Stems, leaves and whole herbs	4	a, b
Taraxaci Herba	Stems, leaves and whole herbs	3	a, b
Chrysanthemi Flos	Flowers	5	b, c
Lonicerae Japonicae Flos	Flowers	18	a, c
Magnoliae Flos	Flowers	3	b, c
Rosae Rugosae Flos	Flowers	3	a, c
Arecae Semen	Fruits and seeds	7	a, c
Armeniacae Semen Amarum	Fruits and seeds	4	b, c
Cassiae Semen	Fruits and seeds	4	a, b
Citri Reticulatae Pericarpium	Fruits and seeds	13	b, c
Crataegi Fructus	Fruits and seeds	13	a, b, c
Gardeniae Fructus	Fruits and seeds	8	a, c
Hordei Fructus Germinatus	Fruits and seeds	4	a, b
Jujubae Fructus	Fruits and seeds	4	a, b
Lycii Fructus	Fruits and seeds	12	b
Myristicae Semen	Fruits and seeds	4	a, b
Nelumbinis Semen	Fruits and seeds	4	a, b, c
Persicae Semen	Fruits and seeds	3	a, b
Schisandrae Chinensis Fructus	Fruits and seeds	5	a, b, c
Sterculiae Lychnophorae Semen	Fruits and seeds	4	a, b
Ziziphi Spinosae Semen	Fruits and seeds	5	a, b
Aspongopus	animal-derived decoction pieces	10	b, c
Bombyx Batryticatus	animal-derived decoction pieces	13	b, c
Cicadae Periostracum	animal-derived decoction pieces	13	b, c
Eupolyphaga Steleophaga	animal-derived decoction pieces	10	a, b, c
Hirudo	animal-derived decoction pieces	13	a, b, c
Pheretima	animal-derived decoction pieces	13	a, b, c
Sepiae Endoconcha	animal-derived decoction pieces	3	a, b, c

Note: Source designations: a, hospitals; b, retail pharmacies; c, herbal medicine markets(online or offline).

Table S5 *The results of the Kruskal－Wallis H test and the pairwise comparisons among the three types of TCMMs.*

PTEs	Kruskal-W	*P*	Sig.	Pairwise comparison results

	allis H			
As	15.013	0.0006	***	stems and leaves, whole herbs, flowers, fruits and seeds VS roots and rhizomes：0.869<br>stems and leaves, whole herbs, flowers, fruits and seeds VS animal-derived decoction pieces：0.0003<br>roots and rhizomes VS animal-derived decoction pieces：0.005
Hg	10.763	0.0046	**	stems and leaves, whole herbs, flowers, fruits and seeds VS roots and rhizomes：0.137<br>stems and leaves, whole herbs, flowers, fruits and seeds VS animal-derived decoction pieces：0.183<br>roots and rhizomes VS animal-derived decoction pieces：0.005
Pb	11.278	0.0036	**	stems and leaves, whole herbs, flowers, fruits and seeds VS roots and rhizomes：0.476<br>stems and leaves, whole herbs, flowers, fruits and seeds VS animal-derived decoction pieces：0.003<br>roots and rhizomes VS animal-derived decoction pieces：0.054
Cd	17.273	0.0002	***	stems and leaves, whole herbs, flowers, fruits and seeds VS roots and rhizomes：0.035<br>stems and leaves, whole herbs, flowers, fruits and seedsVS animal-derived decoction pieces：0.0003<br>roots and rhizomes VS animal-derived decoction pieces：0.079
Cu	12.663	0.0018	**	stems and leaves, whole herbs, flowers, fruits and seeds VS roots and rhizomes：0.061<br>stems and leaves, whole herbs, flowers, fruits and seedsVS animal-derived decoction pieces：0.188<br>roots and rhizomes VS animal-derived decoction pieces：0.003

Table S6 *Single-Factor and Nemerow Pollution Indices of PTEs in TCMMs*

Name	P_{As}	P_{Hg}	P_{Pb}	P_{Cd}	P_{Cu}	P_{N}
Angelicae Dahuricae Radix	0.18	0.08	0.05	0.02	0.56	0.42
Angelicae Sinensis Radix	0.30	0.00	0.11	0.02	0.23	0.23
Astragali Radix	0.14	0.10	0.12	0.01	0.33	0.25
Atractylodis Macrocephalae Rhizoma	0.12	0.00	0.00	0.11	0.58	0.43
Coptidis Rhizoma	0.13	0.26	0.03	0.00	0.79	0.58
Dioscoreae Rhizoma	0.01	0.03	0.14	0.03	0.11	0.11
Gastrodiae Rhizoma	0.02	0.10	0.00	0.15	0.12	0.12
Ginseng Radix et Rhizoma	0.01	0.14	0.02	0.10	0.35	0.26
Ginseng Radix et Rhizoma Rubra	0.03	1.47	0.00	0.05	0.45	1.08

Glycyrrhizae Radix et Rhizoma	0.12	0.10	0.04	0.02	0.42	0.31
Hedysari Radix	0.06	0.09	0.19	0.10	0.47	0.35
Notoginseng Radix et Rhizoma	1.12	0.24	0.01	0.05	0.18	0.82
Paeoniae Radix Alba	0.05	0.09	0.02	0.06	0.35	0.26
Paeoniae Radix Rubra	0.15	0.18	0.01	0.06	0.19	0.16
Panacis Quinquefolii Radix	0.01	0.16	0.01	0.10	0.46	0.34
Pinelliae Rhizoma	0.07	0.13	0.02	0.21	0.13	0.17
Platycodonis Radix	0.43	0.24	0.00	0.00	0.24	0.33
Polygalae Radix	0.22	0.19	0.01	0.01	0.33	0.25
Polygonati Rhizoma	0.10	0.00	0.13	0.45	0.00	0.33
Polygoni Multiflori Radix	0.08	0.33	0.03	0.11	0.20	0.26
Pseudostellariae Radix	0.11	0.22	0.01	0.01	0.21	0.18
Puerariae Lobatae Radix	0.22	0.00	0.16	0.33	0.33	0.28
Rhodiolae Crenulatae Radix et Rhizoma	0.10	0.14	0.51	0.24	0.00	0.39
Salviae Miltiorrhizae Radix et Rhizoma	0.16	0.16	0.14	0.09	0.72	0.54
Arecae Semen	0.01	0.25	0.00	0.01	0.30	0.23
Armeniacae Semen Amarum	0.02	0.11	0.00	0.00	0.47	0.34
Artemisiae Argyi Folium	0.28	3.11	0.00	0.01	0.55	2.27
Blumeae Balsamiferae Herba	0.05	0.17	0.08	0.04	0.25	0.19
Cassiae Semen	0.03	0.17	0.00	0.02	0.55	0.41
Chrysanthemi Flos	0.19	0.44	0.01	0.00	0.73	0.55
Citri Reticulatae Pericarpium	0.17	0.10	0.03	0.01	0.09	0.13
Crataegi Fructus	0.04	0.09	0.11	0.03	0.12	0.10
Dendrobii Caulis	0.19	1.05	0.01	0.00	1.03	0.81
Epimedii Folium	0.41	0.70	0.03	0.08	0.33	0.54
Eriobotryae Folium	0.53	0.78	0.00	0.03	0.57	0.61
Gardeniae Fructus	0.07	0.01	0.09	0.10	0.40	0.30
Ginkgo Folium	0.11	1.44	0.03	0.02	0.49	1.06
Hordei Fructus Germinatus	0.06	0.23	0.02	0.00	0.29	0.23
Isatidis Folium	0.27	1.33	0.02	0.01	0.80	1.00
Jujubae Fructus	0.04	0.13	0.00	0.01	0.19	0.14
Lonicerae Japonicae Flos	0.18	0.27	0.27	0.27	0.67	0.53
Lycii Fructus	0.02	0.07	0.02	0.03	0.37	0.27
Magnoliae Flos	0.14	1.83	0.04	0.07	0.81	1.36
Myristicae Semen	0.03	0.34	0.02	0.01	0.58	0.43
Nelumbinis Semen	0.01	0.10	0.02	0.00	0.67	0.49
Persicae Semen	0.01	0.00	0.00	0.07	0.30	0.22
Platycladi Cacumen	0.00	0.20	0.01	0.01	1.08	0.78
Rosae Rugosae Flos	0.03	0.08	0.00	0.04	0.18	0.14
Schisandrae Chinensis Fructus	0.05	0.33	0.01	0.00	0.33	0.26
Sterculiae Lychnophorae Semen	0.00	0.17	0.00	0.00	0.46	0.34
Taraxaci Herba	0.17	0.11	0.49	0.50	0.58	0.49
Ziziphi Spinosae Semen	0.02	0.12	0.00	0.00	0.54	0.39
Aspongopus	0.72	0.59	0.69	0.16	0.80	0.71

Bombyx Batryticatus	0.71	0.61	2.16	0.41	0.40	1.64
Cicadae Periostracum	1.13	0.23	1.14	0.32	0.37	0.92
Eupolyphaga Steleophaga	0.57	0.52	0.28	0.14	1.15	0.90
Hirudo	0.69	0.26	0.26	0.06	0.96	0.75
Pheretima	4.16	1.23	0.57	1.12	0.63	3.14
Sepiae Endoconcha	0.03	0.22	0.00	0.04	0.90	0.66

Table S7 *Single-factor pollution index Table*

Name	Pollution factors	Single-factor pollution index	Pollution Level	Name	Pollution factors	Single-factor pollution index	Pollution Level
Pheretima	As	4.16	Heavy pollution	*Pheretima*	Hg	1.23	Slight pollution
Cicadae Periostracum	As	1.13	Slight pollution	*Dendrobii Caulis*	Hg	1.05	Slight pollution
Notoginseng Radix et Rhizoma	As	1.12	Slight pollution	*Bombyx Batryticatus*	Pb	2.16	Moderate pollution
Artemisiae Argyi Folium	Hg	3.11	Heavy pollution	*Cicadae Periostracum*	Pb	1.14	Slight pollution
Magnoliae Flos	Hg	1.83	Slight pollution	*Pheretima*	Cd	1.12	Slight pollution
Ginseng Radix et Rhizoma Rubra	Hg	1.47	Slight pollution	*Eupolyphaga Steleophaga*	Cu	1.15	Slight pollution
Ginkgo Folium	Hg	1.44	Slight pollution	*Platycladi Cacumen*	Cu	1.08	Slight pollution
Isatidis Folium	Hg	1.33	Slight pollution	*Dendrobii Caulis*	Cu	1.03	Slight pollution

Table S8 *Nemerow Pollution Index Table*

Name	Pollution factors	Nemerow Pollution Index	Pollution Level	Name	Pollution factors	Nemerow Pollution Index	Pollution Level
Pheretima	As	3.14	Heavy pollution	*Dendrobii Caulis*	Hg	0.81	Alert level
Notoginseng Radix et Rhizoma	As	0.82	Alert level	*Bombyx Batryticatus*	Pb	1.64	Slight pollution
Artemisiae Argyi Folium	Hg	2.27	Moderate pollution	*Cicadae Periostracum*	Pb	0.92	Alert level
Magnoliae Flos	Hg	1.36	Slight pollution	*Eupolyphaga Steleophaga*	Cu	0.90	Alert level
Ginseng Radix et Rhizoma Rubra	Hg	1.08	Slight pollution	*Platycladi Cacumen*	Cu	0.78	Alert level

Ginkgo Folium	Hg	1.06	Slight pollution	*Hirudo*	Cu	0.75	Alert level
Isatidis Folium	Hg	1.00	Slight pollution	*Aspongopus*	Cu	0.71	Alert level

Table S9-1 *Target hazard quotient index and hazard index of roots and rhizomes*

Name	THQ_{AS}	THQ_{Hg}	THQ_{Pb}	THQ_{Cd}	THQ_{Cu}	HI
Angelicae Dahuricae Radix	4.94×10^{-3}	1.52×10^{-4}	1.46×10^{-3}	9.81×10^{-5}	4.60×10^{-4}	7.11×10^{-3}
Angelicae Sinensis Radix	9.77×10^{-3}	1.69×10^{-7}	3.73×10^{-3}	1.49×10^{-4}	2.27×10^{-4}	1.39×10^{-2}
Astragali Radix	1.16×10^{-2}	5.81×10^{-4}	1.01×10^{-2}	2.30×10^{-4}	8.13×10^{-4}	2.34×10^{-2}
Atractylodis Macrocephalae Rhizoma	4.00×10^{-3}	1.69×10^{-7}	3.95×10^{-7}	7.45×10^{-4}	5.75×10^{-4}	5.32×10^{-3}
Coptidis Rhizoma	1.81×10^{-3}	2.47×10^{-4}	3.03×10^{-5}	1.23×10^{-9}	3.23×10^{-4}	2.41×10^{-3}
Dioscoreae Rhizoma	9.26×10^{-4}	1.66×10^{-4}	9.76×10^{-4}	1.36×10^{-4}	2.78×10^{-4}	2.48×10^{-3}
Gastrodiae Rhizoma	4.28×10^{-4}	1.92×10^{-4}	3.29×10^{-5}	7.95×10^{-4}	9.61×10^{-5}	1.54×10^{-3}
Ginseng Radix et Rhizoma	1.95×10^{-4}	2.28×10^{-4}	4.96×10^{-4}	5.11×10^{-4}	2.58×10^{-4}	1.69×10^{-3}
Ginseng Radix et Rhizoma Rubra	6.17×10^{-4}	2.49×10^{-3}	2.96×10^{-7}	2.22×10^{-4}	3.33×10^{-4}	3.66×10^{-3}
Glycyrrhizae Radix et Rhizoma	3.29×10^{-3}	1.95×10^{-4}	1.14×10^{-3}	1.13×10^{-4}	3.44×10^{-4}	5.08×10^{-3}
Hedysari Radix	4.80×10^{-3}	5.00×10^{-4}	1.53×10^{-2}	1.64×10^{-3}	1.15×10^{-3}	2.34×10^{-2}
Notoginseng Radix et Rhizoma	2.76×10^{-2}	3.97×10^{-4}	1.38×10^{-4}	2.40×10^{-4}	1.35×10^{-4}	2.85×10^{-2}
Paeoniae Radix Alba	1.86×10^{-3}	2.58×10^{-4}	6.62×10^{-4}	5.17×10^{-4}	4.31×10^{-4}	3.73×10^{-3}
Paeoniae Radix Rubra	5.00×10^{-3}	4.00×10^{-4}	3.79×10^{-5}	1.22×10^{-4}	1.88×10^{-4}	5.75×10^{-3}
Panacis Quinquefolii Radix	2.24×10^{-4}	1.77×10^{-4}	1.83×10^{-4}	3.20×10^{-4}	2.26×10^{-4}	1.13×10^{-3}
Pinelliae Rhizoma	1.83×10^{-3}	2.20×10^{-4}	4.27×10^{-5}	3.05×10^{-4}	9.78×10^{-5}	2.50×10^{-3}
Platycodonis Radix	1.18×10^{-2}	4.41×10^{-4}	6.16×10^{-6}	2.85×10^{-6}	1.94×10^{-4}	1.24×10^{-2}
Polygalae Radix	6.02×10^{-3}	3.57×10^{-4}	1.69×10^{-5}	1.68×10^{-5}	2.68×10^{-4}	6.68×10^{-3}
Polygonati Rhizoma	3.95×10^{-3}	2.11×10^{-7}	4.70×10^{-4}	1.10×10^{-3}	6.16×10^{-7}	5.52×10^{-3}
Polygoni Multiflori Radix	1.37×10^{-3}	3.70×10^{-4}	3.73×10^{-5}	1.05×10^{-4}	1.01×10^{-4}	1.98×10^{-3}

Pseudostellariae Radix	9.28×10^{-3}	1.26×10^{-3}	8.66×10^{-5}	4.19×10^{-5}	5.16×10^{-4}	1.12×10^{-2}
Puerariae Lobatae Radix	8.84×10^{-3}	2.11×10^{-7}	6.58×10^{-3}	2.71×10^{-3}	4.01×10^{-4}	1.85×10^{-2}
Rhodiolae Crenulatae Radix et Rhizoma	1.56×10^{-3}	1.56×10^{-4}	8.38×10^{-3}	7.73×10^{-4}	2.47×10^{-7}	1.09×10^{-2}
Salviae Miltiorrhizae Radix et Rhizoma	6.50×10^{-3}	4.48×10^{-4}	5.62×10^{-3}	7.45×10^{-4}	8.92×10^{-4}	1.42×10^{-2}
Min	1.95×10^{-4}	1.69×10^{-7}	2.96×10^{-7}	1.23×10^{-9}	2.47×10^{-7}	1.13×10^{-3}
Median	3.97×10^{-3}	2.37×10^{-4}	3.26×10^{-4}	2.35×10^{-4}	2.73×10^{-4}	5.63×10^{-3}
Max	2.76×10^{-2}	2.49×10^{-3}	1.53×10^{-2}	2.71×10^{-3}	1.15×10^{-3}	2.85×10^{-2}

Table S9-2 *Target hazard quotient index and hazard index of stems and leaves, whole herbs, flowers, fruits and seeds*

Name	THQ_{AS}	THQ_{Hg}	THQ_{Pb}	THQ_{Cd}	THQ_{Cu}	HI
Arecae Semen	2.49×10^{-4}	4.63×10^{-4}	2.96×10^{-6}	1.28×10^{-5}	2.50×10^{-4}	9.78×10^{-4}
Armeniacae Semen Amarum	5.31×10^{-4}	1.97×10^{-4}	5.34×10^{-6}	1.15×10^{-6}	3.85×10^{-4}	1.12×10^{-3}
Artemisiae Argyi Folium	6.84×10^{-3}	5.26×10^{-3}	1.67×10^{-6}	1.47×10^{-5}	4.08×10^{-4}	1.25×10^{-2}
Blumeae Balsamiferae Herba	2.06×10^{-3}	4.89×10^{-4}	2.70×10^{-4}	9.70×10^{-5}	3.05×10^{-4}	3.22×10^{-3}
Cassiae Semen	1.12×10^{-3}	4.68×10^{-4}	6.36×10^{-6}	5.67×10^{-5}	6.82×10^{-4}	2.33×10^{-3}
Chrysanthemi Flos	5.28×10^{-3}	8.23×10^{-4}	1.35×10^{-5}	7.23×10^{-6}	6.04×10^{-4}	6.73×10^{-3}
Citri Reticulatae Pericarpium	4.75×10^{-3}	1.80×10^{-4}	6.89×10^{-5}	1.13×10^{-5}	7.44×10^{-5}	5.08×10^{-3}
Crataegi Fructus	1.16×10^{-3}	1.93×10^{-4}	3.07×10^{-4}	5.51×10^{-5}	1.19×10^{-4}	1.83×10^{-3}
Dendrobii Caulis	6.37×10^{-3}	2.38×10^{-3}	2.31×10^{-5}	6.26×10^{-6}	1.02×10^{-3}	9.79×10^{-3}
Epimedii Folium	1.13×10^{-2}	1.31×10^{-3}	7.80×10^{-5}	1.25×10^{-4}	2.71×10^{-4}	1.30×10^{-2}
Eriobotryae Folium	1.46×10^{-2}	1.46×10^{-3}	6.83×10^{-6}	4.70×10^{-5}	4.66×10^{-4}	1.66×10^{-2}
Gardeniae Fructus	1.90×10^{-3}	1.66×10^{-5}	2.17×10^{-4}	1.62×10^{-4}	3.30×10^{-4}	2.63×10^{-3}
Ginkgo Folium	3.70×10^{-3}	3.25×10^{-3}	9.82×10^{-5}	3.07×10^{-5}	4.79×10^{-4}	7.56×10^{-3}
Hordei Fructus Germinatus	2.61×10^{-3}	6.48×10^{-4}	5.86×10^{-5}	3.70×10^{-9}	3.63×10^{-4}	3.68×10^{-3}
Isatidis Folium	1.12×10^{-2}	3.76×10^{-3}	6.24×10^{-5}	3.12×10^{-5}	9.89×10^{-4}	1.61×10^{-2}

Jujubae Fructus	1.77×10^{-3}	3.66×10^{-4}	1.29×10^{-5}	1.30×10^{-5}	2.35×10^{-4}	2.40×10^{-3}
Lonicerae Japonicae Flos	7.23×10^{-3}	7.52×10^{-4}	9.60×10^{-4}	6.70×10^{-4}	8.32×10^{-4}	1.04×10^{-2}
Lycii Fructus	5.49×10^{-4}	1.48×10^{-4}	4.73×10^{-5}	6.83×10^{-5}	3.64×10^{-4}	1.18×10^{-3}
Magnoliae Flos	3.88×10^{-3}	3.44×10^{-3}	8.35×10^{-5}	1.07×10^{-4}	6.66×10^{-4}	8.18×10^{-3}
Myristicae Semen	8.32×10^{-4}	6.34×10^{-4}	4.12×10^{-5}	1.66×10^{-5}	4.76×10^{-4}	2.00×10^{-3}
Nelumbinis Semen	5.04×10^{-4}	2.68×10^{-4}	6.37×10^{-5}	3.27×10^{-6}	8.25×10^{-4}	1.66×10^{-3}
Persicae Semen	2.29×10^{-4}	1.41×10^{-7}	2.82×10^{-8}	1.10×10^{-4}	2.48×10^{-4}	5.86×10^{-4}
Platycladi Cacumen	8.22×10^{-7}	4.54×10^{-4}	1.62×10^{-5}	2.30×10^{-5}	1.06×10^{-3}	1.56×10^{-3}
Rosae Rugosae Flos	5.48×10^{-4}	8.49×10^{-5}	1.69×10^{-8}	4.27×10^{-5}	9.05×10^{-5}	7.66×10^{-4}
Schisandrae Chinensis Fructus	8.98×10^{-4}	3.72×10^{-4}	1.16×10^{-5}	2.17×10^{-6}	1.63×10^{-4}	1.45×10^{-3}
Sterculiae Lychnophorae Semen	6.85×10^{-7}	3.22×10^{-4}	8.55×10^{-6}	4.13×10^{-7}	3.75×10^{-4}	7.07×10^{-4}
Taraxaci Herba	6.85×10^{-3}	3.19×10^{-4}	1.71×10^{-3}	1.22×10^{-3}	7.19×10^{-4}	1.08×10^{-2}
Ziziphi Spinosae Semen	1.02×10^{-3}	3.30×10^{-4}	3.22×10^{-6}	2.47×10^{-6}	6.60×10^{-4}	2.02×10^{-3}
Min	6.85×10^{-7}	1.41×10^{-7}	1.69×10^{-8}	3.70×10^{-9}	7.44×10^{-5}	5.86×10^{-4}
Median	1.84×10^{-3}	4.58×10^{-4}	3.21×10^{-5}	2.68×10^{-5}	3.96×10^{-4}	2.51×10^{-3}
Max	1.46×10^{-2}	5.26×10^{-3}	1.71×10^{-3}	1.22×10^{-3}	1.06×10^{-3}	1.66×10^{-2}

Table S9-3 *Target hazard quotient index and hazard index of animal-derived decoction pieces.*

Name	THQ_{AS}	THQ_{Hg}	THQ_{Pb}	THQ_{Cd}	THQ_{Cu}	*HI*
Aspongopus	1.78×10^{-2}	1.00×10^{-3}	1.47×10^{-3}	2.33×10^{-4}	5.93×10^{-4}	2.11×10^{-2}
Bombyx Batryticatus	1.95×10^{-2}	1.14×10^{-3}	5.08×10^{-3}	6.80×10^{-4}	3.26×10^{-4}	2.67×10^{-2}
Cicadae Periostracum	1.86×10^{-2}	2.54×10^{-4}	1.60×10^{-3}	3.12×10^{-4}	1.80×10^{-4}	2.09×10^{-2}
Eupolyphaga Steleophaga	1.39×10^{-2}	8.76×10^{-4}	5.99×10^{-4}	2.00×10^{-4}	8.52×10^{-4}	1.65×10^{-2}
Hirudo	1.42×10^{-2}	7.39×10^{-4}	3.68×10^{-4}	3.20×10^{-5}	2.37×10^{-4}	1.56×10^{-2}
Pheretima	1.14×10^{-1}	2.31×10^{-3}	1.33×10^{-3}	1.84×10^{-3}	5.17×10^{-4}	1.20×10^{-1}

Sepiae Endoconcha	3.56×10^{-3}	4.07×10^{-4}	2.82×10^{-8}	3.01×10^{-4}	7.43×10^{-4}	5.01×10^{-3}
Min	3.56×10^{-3}	2.54×10^{-4}	2.82×10^{-8}	3.20×10^{-5}	1.80×10^{-4}	5.01×10^{-3}
Median	1.78×10^{-2}	8.76×10^{-4}	1.33×10^{-3}	3.01×10^{-4}	5.17×10^{-4}	2.09×10^{-2}
Max	1.14×10^{-1}	2.31×10^{-3}	5.08×10^{-3}	1.84×10^{-3}	8.52×10^{-4}	1.20×10^{-1}

Tabe S10-1 *Carcinogenic risk index of roots and rhizomes*

Name	CR-AS	CR-Pb	CR-Cd
Angelicae Dahuricae Radix	2.22×10^{-6}	3.72×10^{-9}	1.80×10^{-7}
Angelicae Sinensis Radix	4.40×10^{-6}	9.50×10^{-9}	2.73×10^{-7}
Astragali Radix	5.23×10^{-6}	2.58×10^{-8}	4.20×10^{-7}
Atractylodis Macrocephalae Rhizoma	1.80×10^{-6}	1.01×10^{-12}	1.36×10^{-6}
Coptidis Rhizoma	8.14×10^{-7}	9.00×10^{-10}	7.52×10^{-12}
Dioscoreae Rhizoma	4.17×10^{-7}	2.90×10^{-8}	8.27×10^{-7}
Gastrodiae Rhizoma	1.93×10^{-7}	8.40×10^{-11}	1.46×10^{-6}
Ginseng Radix et Rhizoma	8.78×10^{-8}	1.27×10^{-9}	9.36×10^{-7}
Ginseng Radix et Rhizoma Rubra	2.77×10^{-7}	7.55×10^{-13}	4.06×10^{-7}
Glycyrrhizae Radix et Rhizoma	1.48×10^{-6}	2.91×10^{-9}	2.06×10^{-7}
Hedysari Radix	2.16×10^{-6}	3.91×10^{-8}	3.01×10^{-6}
Notoginseng Radix et Rhizoma	1.24×10^{-5}	3.51×10^{-10}	4.39×10^{-7}
Paeoniae Radix Alba	8.37×10^{-7}	1.69×10^{-9}	9.46×10^{-7}
Paeoniae Radix Rubra	2.25×10^{-6}	1.13×10^{-9}	7.45×10^{-7}
Panacis Quinquefolii Radix	1.01×10^{-7}	4.67×10^{-10}	5.85×10^{-7}
Pinelliae Rhizoma	8.25×10^{-7}	1.27×10^{-9}	1.86×10^{-6}
Platycodonis Radix	5.31×10^{-6}	1.83×10^{-10}	1.74×10^{-8}
Polygalae Radix	2.71×10^{-6}	5.04×10^{-10}	1.03×10^{-7}
Polygonati Rhizoma	1.78×10^{-6}	1.40×10^{-8}	6.71×10^{-6}
Polygoni Multiflori Radix	6.15×10^{-7}	1.11×10^{-9}	6.41×10^{-7}
Pseudostellariae Radix	4.17×10^{-6}	2.58×10^{-9}	2.56×10^{-7}
Puerariae Lobatae Radix	3.98×10^{-6}	1.68×10^{-8}	4.96×10^{-6}
Rhodiolae Crenulatae Radix et Rhizoma	7.03×10^{-7}	2.14×10^{-8}	1.41×10^{-6}
Salviae Miltiorrhizae Radix et Rhizoma	2.92×10^{-6}	1.43×10^{-8}	1.36×10^{-6}
Min	8.78×10^{-8}	7.55×10^{-13}	7.52×10^{-12}
Median	1.79×10^{-6}	1.48×10^{-9}	6.93×10^{-7}
Max	1.24×10^{-5}	3.91×10^{-8}	6.71×10^{-6}

Table S10-2 *Carcinogenic risk index of stems and leaves, whole herbs, flowers, fruits and seeds*

Name	CR-AS	CR-Pb	CR-Cd
Arecae Semen	1.12×10^{-6}	8.81×10^{-11}	7.81×10^{-8}
Armeniacae Semen Amarum	2.39×10^{-7}	1.59×10^{-10}	7.03×10^{-9}
Artemisiae Argyi Folium	3.08×10^{-6}	4.97×10^{-11}	8.96×10^{-8}
Blumeae Balsamiferae Herba	9.25×10^{-7}	8.03×10^{-9}	5.92×10^{-7}
Cassiae Semen	5.04×10^{-7}	1.89×10^{-10}	3.46×10^{-7}
Chrysanthemi Flos	2.38×10^{-6}	4.01×10^{-10}	4.41×10^{-8}
Citri Reticulatae Pericarpium	2.14×10^{-6}	2.05×10^{-9}	6.87×10^{-8}
Crataegi Fructus	5.21×10^{-7}	9.13×10^{-9}	3.36×10^{-7}
Dendrobii Caulis	2.86×10^{-6}	6.87×10^{-10}	3.82×10^{-8}

Epimedii Folium	5.07×10^{-6}	2.32×10^{-9}	7.64×10^{-7}
Eriobotryae Folium	6.59×10^{-6}	2.03×10^{-10}	2.87×10^{-7}
Gardeniae Fructus	8.55×10^{-7}	6.46×10^{-9}	9.89×10^{-7}
Ginkgo Folium	1.67×10^{-6}	2.92×10^{-9}	1.87×10^{-7}
Hordei Fructus Germinatus	1.17×10^{-6}	1.74×10^{-9}	2.26×10^{-11}
Isatidis Folium	5.06×10^{-6}	1.86×10^{-9}	1.90×10^{-7}
Jujubae Fructus	7.98×10^{-7}	3.84×10^{-10}	7.94×10^{-8}
Lonicerae Japonicae Flos	3.25×10^{-6}	2.86×10^{-8}	4.09×10^{-6}
Lycii Fructus	2.47×10^{-7}	1.41×10^{-9}	4.16×10^{-7}
Magnoliae Flos	1.75×10^{-6}	2.48×10^{-9}	6.52×10^{-7}
Myristicae Semen	3.74×10^{-7}	1.23×10^{-9}	1.02×10^{-7}
Nelumbinis Semen	2.27×10^{-7}	1.89×10^{-9}	1.99×10^{-8}
Persicae Semen	1.03×10^{-7}	8.38×10^{-13}	6.68×10^{-7}
Platycladi Cacumen	3.70×10^{-10}	4.82×10^{-10}	1.40×10^{-7}
Rosae Rugosae Flos	2.47×10^{-7}	5.03×10^{-13}	2.61×10^{-7}
Schisandrae Chinensis Fructus	4.04×10^{-7}	3.44×10^{-10}	1.32×10^{-8}
Sterculiae Lychnophorae Semen	3.08×10^{-10}	2.55×10^{-10}	2.52×10^{-9}
Taraxaci Herba	3.08×10^{-6}	5.10×10^{-8}	7.47×10^{-6}
Ziziphi Spinosae Semen	4.59×10^{-7}	9.57×10^{-11}	1.51×10^{-8}
Min	3.08×10^{-10}	5.03×10^{-13}	2.26×10^{-11}
Median	8.90×10^{-7}	9.56×10^{-10}	1.64×10^{-7}
Max	6.59×10^{-6}	5.10×10^{-8}	7.47×10^{-6}

Table S10-3 *Carcinogenic risk index of animal-derived decoction pieces.*

Name	CR-AS	CR-Pb	CR-Cd
Aspongopus	8.01×10^{-6}	4.36×10^{-8}	1.42×10^{-6}
Bombyx Batryticatus	8.77×10^{-6}	1.51×10^{-7}	4.15×10^{-6}
Cicadae Periostracum	8.35×10^{-6}	4.77×10^{-8}	1.90×10^{-6}
Eupolyphaga Steleophaga	6.27×10^{-6}	1.78×10^{-8}	1.22×10^{-6}
Hirudo	6.38×10^{-6}	1.10×10^{-8}	1.95×10^{-7}
Pheretima	5.13×10^{-5}	3.96×10^{-8}	1.12×10^{-5}
Sepiae Endoconcha	1.60×10^{-6}	8.38×10^{-13}	1.84×10^{-6}
Min	1.60×10^{-6}	8.38×10^{-13}	1.95×10^{-7}
Median	8.01×10^{-6}	3.96×10^{-8}	1.84×10^{-6}
Max	5.13×10^{-5}	1.51×10^{-7}	1.12×10^{-5}

Table S11 *Details of the parameters for the health risk assessment model*

Parameter	Symbol	Value / Category	Distribution Type	Unit
PTEs concentration	C_i (As)	roots and rhizomes:(Mean=0.3525, Std=0.5809)	Log-normal	mg/kg
		stems and leaves, whole herbs, flowers, fruits and seeds:(Mean=0.4079, Std=1.7297)	Log-normal	mg/kg
		animal-derived decoction pieces:(Min=0.26, max=8.3224)	Uniform	mg/kg
	C_i (Hg)	roots and rhizomes:(Mean=0.0531, Std=0.1524)	Log-normal	mg/kg
		stems and leaves, whole herbs, flowers, fruits and seeds:(Mean=0.1033, Std=0.2050)	Log-normal	mg/kg
		animal-derived decoction pieces:(Min=0.0433, max=0.2622)	Uniform	mg/kg
	C_i (Pb)	roots and rhizomes:(Mean=0.8448, Std=6.6760)	Normal	mg/kg
		stems and leaves, whole herbs, flowers, fruits and seeds:(Mean=0.3214, Std=2.0765)	Log-normal	mg/kg
		animal-derived decoction pieces:(Min=0.0001, max=10.8093)	Uniform	mg/kg
	C_i (Cd)	roots and rhizomes:(Mean=0.1122, Std=0.2139)	Log-normal	mg/kg
		stems and leaves, whole herbs, flowers, fruits and seeds:(Mean=0.0773, Std=0.5142)	Log-normal	mg/kg
		animal-derived decoction pieces:(Min=0.0648, max=1.1181)	Uniform	mg/kg
	C_i (Cu)	roots and rhizomes:(Mean=6.4515, Std=4.1177)	Normal	mg/kg
		stems and leaves, whole herbs, flowers, fruits and seeds:(Mean=9.8486, Std=5.1605)	Log-normal	mg/kg
		animal-derived decoction pieces:(Min=7.3155, max=23.0408)	Uniform	mg/kg
Daily ingestion rate	IR	roots and rhizomes:(Mean=9.75, Std=7.01)	Log-normal	g/day
		stems and leaves, whole herbs, flowers, fruits and seeds:(Mean=8.75, Std=2.45)	Log-normal	g/day
		animal-derived decoction pieces:(Min=2, max=7.5)	Uniform	g/day

Exposure frequency	EF	(Min=30, max=90)	Uniform	days/year
Exposure duration	ED	20	Constant	years
Body weight	BW	Mean=60, Std=8	Normal	kg
Average time	AT	25550	Constant	days
	t(As)	0.35	Constant	——
	t(Hg)	0.24	Constant	——
Transfer rate	t(Pb)	0.14	Constant	——
	t(Cd)	0.14	Constant	——
	t(Cu)	0.14	Constant	——
	RfD(As)	0.0003	Constant	$mg \cdot kg^{-1} \cdot d^{-1}$
	RfD(Hg)	0.0003	Constant	$mg \cdot kg^{-1} \cdot d^{-1}$
Reference dose	RfD(Pb)	0.0035	Constant	$mg \cdot kg^{-1} \cdot d^{-1}$
	RfD(Cd)	0.001	Constant	$mg \cdot kg^{-1} \cdot d^{-1}$
	RfD(Cu)	0.04	Constant	$mg \cdot kg^{-1} \cdot d^{-1}$
	CSF(As)	1.5	Constant	$mg \cdot kg^{-1} \cdot d^{-1}$
Carcinogenic slope factor	CSF(Pb)	0.0085	Constant	$mg \cdot kg^{-1} \cdot d^{-1}$
	CSF(Cd)	6.1	Constant	$mg \cdot kg^{-1} \cdot d^{-1}$

Table S12 *Summary of Monte Carlo simulation results*

risk indicator	roots and rhizomes	stems and leaves, whole herbs, flowers, fruits and seeds	animal-derived decoction pieces
HI Mean Value	4.09×10^{-3}	3.99×10^{-3}	2.07×10^{-2}
HI-95th percentile	1.39×10^{-2}	1.42×10^{-2}	5.04×10^{-2}
p(HI>1)	0%	0%	0%
CR Mean Value	2.18×10^{-6}	1.82×10^{-6}	1.04×10^{-5}
CR-95th percentile	7.55×10^{-6}	7.14×10^{-6}	2.48×10^{-5}
p(CR>1E-06)	53.78%	33.57%	99.18%
p(CR>1E-04)	0%	0.05%	0%
